# Single‐Pixel Infrared Miniaturized Spectrometer Enabled by Ultra‐Broadband Reconfigurable Photodetection

**DOI:** 10.1002/advs.202500830

**Published:** 2025-04-02

**Authors:** Wenyue Liang, Xianghong Nan, Wenfeng Cai, Ning Tan, Qilin Zheng, Yuyao Lu, Yongyue Huang, Jiahao Yan, Dangyuan Lei, Long Wen, Yanjun Liu, Qin Chen

**Affiliations:** ^1^ Guangdong Provincial Key Laboratory of Nanophotonic Manipulation Institute of Nanophotonics College of Physics & Optoelectronic Engineering Jinan University Guangzhou 511443 China; ^2^ Department of Electrical and Electronic Engineering Southern University of Science and Technology Shenzhen 518055 China; ^3^ Department of Materials Science and Engineering City University of Hong Kong Kowloon Hong Kong 999077 China

**Keywords:** liquid crystal, modulation, photodetection, sensing, spectrometer

## Abstract

Miniaturized spectrometers utilizing a single reconfigurable photodetector (PD) are highly attractive in the infrared (IR) range due to their advantages in terms of cost, ease of integration, and reduced system complexity. However, such devices usually suffer from either limited wavelength tuning range or high spectral correlation in spectral sampling. Here, a new concept based on tunable guided mode resonances and surface plasmon resonances in a simple liquid crystal/Au stack is proposed to break the wavelength tuning range limit and simultaneously enable low spectral correlation. A chip‐scale IR spectrometer using a single‐pixel PbS PD is realized with a remarkably large wavelength tuning range over 850 nm (1150–2000 nm) and high fidelity (mean square error ≈0.001) in spectrum measurement. Such a novel technique is applied in plastic sorting and demonstrated remarkable improvement in sorting accuracy benefiting from its broadband property and distinct spectral responses at various sampling biases. Furthermore, a post‐tuned operating mode for efficient and accurate spectroscopy is demonstrated by customizing the wavelength/bias scanning strategies, demonstrating the high flexibility of this technology. Full‐vector analysis considering the interface anchoring effect and the anisotropic gradient refractive index distribution of liquid crystal is conducted to reveal the fundamental principles of broadband light modulation.

## Graphical abstract

A near‐infrared tunable micro‐spectrometer is achieved by integrating a PbS photodetection unit and a light modulation unit based on liquid crystal tuned optical resonances, demonstrating a wavelength tuning range exceeding 850 nm. This micro‐spectrometer shows high fidelity in spectral reconstruction and is also successfully applied to plastic sorting, achieving 100% accuracy.

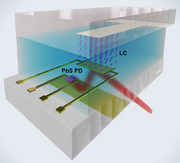

## 1 Introduction

Optical spectrometry is one of the most significant and widely used characterization tools in scientific research and industrial applications.^[^ ^1^ ^]^ Conventional benchtop spectrometers are based on either diffraction gratings or interferometers, both of which rely on bulky dispersive optics and long optical path lengths. All these impede the applications such as on‐site inspection and point‐of‐care diagnosis.^[^ ^2^, ^3^, ^4^ ^]^ Recently, the miniaturization of optical spectrometers has become more popular due to the increasing need for in‐line, in‐the‐field, and point‐of‐use measurements, where a wide variety of design and working principles have been explored including miniaturized dispersive optics,^[^ ^5^, ^6^ ^]^ narrowband filters^[^ ^7^, ^8^, ^9^, ^10^, ^11^, ^12^ ^]^ and computational spectral reconstruction.^[^ ^13^, ^14^, ^15^ ^]^ A photodetector (PD) array is a fundamental component in all these cases. Benefiting from the high maturity of silicon processing and low‐cost image sensors, remarkable progress in integrated spectrometers has been made in visible and short‐wave near‐infrared. Wang et al. presented a 128‐channel Fabry‐Perot (FP) filter integrated micro‐spectrometer operating between 722 and 880 nm with a spectral resolution beyond 6 nm.^[^ ^7^ ^]^ Bao et al. demonstrated a colloidal quantum dot (CQD) micro‐spectrometer by depositing 195 different CQD filters on a charge coupled device, where a spectral resolution ≈3 nm was realized between 390 and 690 nm.^[^ ^8^ ^]^ A sub‐1 nm spectral resolution was reported by Xiong et al. in a spectral imaging chip with 400 spectral channels based on a metasurface covering a wavelength range of 450–750 nm.^[^ ^10^ ^]^ As seen, hundreds of spectral channels are required to obtain high spectral accuracy and large bandwidth, which however increases the complexity of the devices and greatly degrades the signal‐to‐noise ratio. Although optical scattering, routing, and filtering can all be used to construct the dispersion channels,^[^ ^16^, ^17^, ^18^, ^19^, ^20^ ^]^ the fabrication of a multilayer stack or various nanostructures on a large scale is still a great challenge. All these significantly limit the progress of miniaturized spectrometers in long‐wave near‐infrared or mid‐infrared. The high cost of infrared focal plane arrays and technical complexity in monolithic integration of spectral filter arrays limit the applicability for portable applications, although this part of the electromagnetic spectrum is especially interesting for spectral sensing of rotational and vibrational motions of molecules.

Generally, more spectral channels usually provide improved spectral sensing performance. There is a contradiction between the cost and the performance. There are two potential ways to address this issue. One is to reduce the number of spectral channels and apply advanced analysis algorithms to compensate for the limited hardware performance. For example, most recently deep learning (DL) algorithms were adopted to enhance the spectral sensing accuracy of a near‐infrared (NIR) spectral sensor consisting of InGaAs PDs integrated with plasmonic filters down to 3–5 spectral channels.^[^ ^12^ ^]^ Alternatively, a single PD with a reconfigurable spectral response or a chip‐scale interferometer is expected to achieve on‐chip spectroscopy. In this case, spectrometry is realized by sampling the spectral information in time sequence via tuning the spectral response of the PD. Compared to the micro‐spectrometers based on a detector array, such tunable micro‐spectrometers have lower cost, higher signal‐to‐noise ratio, and can be post‐tuned after manufacturing for different application scenarios. There are two options for spectral reconfigurable photodetection. One method is to tune the active materials directly by electrical, thermal, and chemical excitation.^[^ ^21^, ^22^, ^23^, ^24^, ^25^, ^26^, ^27^ ^]^ Guo et al. presented a single‐dot spectrometer utilizing an in situ modulated perovskite PD,^[^ ^22^ ^]^ where a tunable photogain was realized by controlling ion redistribution in the perovskite film through the introduction of LiCl additive. Deng et al. reported a tunable spectrometer based on electrically gated Wse_2_/ReS_2_ heterojunction.^[^ ^23^ ^]^ Tian et al. demonstrated on‐chip spectroscopy with electrochromic modulation.^[^ ^26^ ^]^ Generally, these techniques suffer from high spectral correlations at different sampling biases. For example, the wavelength shift of the peak photoelectric response of the perovskite devices is less than 10 nm,^[^ ^22^ ^]^ only amplitude variations of the ripples are observed in the transmission spectra of electrochromic devices,^[^ ^26^ ^]^ the photoelectric response spectrum of a van der Waals junction remained unchanged even within certain bias voltage ranges.^[^ ^21^ ^]^ Insufficient band structure modulation makes this scheme challenging to achieve high‐resolution spectrometry. The other method is to integrate a spectral tuning unit with a PD,^[^ ^28^, ^29^, ^30^, ^31^, ^32^, ^33^, ^34^, ^35^, ^36^, ^37^, ^38^, ^39^, ^40^, ^41^, ^42^ ^]^ where wavelength selection and photodetection can be optimized separately. The spectral tuning unit usually consists of a resonator and active material. Based on the thermo‐optical effect, Zhang et al. demonstrated a resonant cavity‐enhanced InGaAs PD with a linewidth of 6 nm, where the resonant wavelength can be tuned from 1557 to 1572 nm,^[^ ^36^ ^]^ and Liu et al. demonstrated a tunable Mach‐Zehnder interferometer based chip‐scale Fourier transform spectrometer with a bandwidth of 90 nm and a spectral resolution to 0.47 nm.^[^ ^37^ ^]^ Using a microelectromechanical systems FP cavity, Chang‐Hasnain et al. demonstrated a tunable PD with a spectral width of 1.2 nm in a wavelength tuning range of 33.5 nm.^[^ ^38^ ^]^ The liquid crystal (LC) technique is also widely used for light modulation. Levallois et al. demonstrated an LC‐based tunable InGaAs PD,^[^ ^41^ ^]^ where a Fabry‐Perot cavity was formed on top of the PD by embedding an LC layer between two distributed Bragg reflectors (DBRs) with a 100 nm resonance wavelength tuning range in the C band. Yang et al. incorporated LC atop a silicon metasurface and realized a spectropolarimeter,^[^ ^42^ ^]^ while the wavelength tuning range was limited to 20 nm. Although the resonator‐integrated NIR PDs may enable finer spectral tuning and thus higher spectral resolution, they usually suffer a limited wavelength tuning range as summarized in Table S1 (Supporting Information) due to small dispersion of optical mechanisms, low modulating efficiency or weak interaction between light and active materials, which limits the practical applications.

In this paper, an IR miniaturized spectrometer operating in a scanning mode was demonstrated based on a single reconfigurable PD with both a remarkably large wavelength tuning range over 850 nm and a high spectral fidelity (mean square error, MSE∼0.001). The interaction between full‐vector fields and anisotropic LC molecules was investigated to reveal the fundamental principles of light modulation. Large dispersion and strong electromagnetic field localization of prism‐coupled surface plasmon resonance (SPR) and guided mode resonance (GMR) contribute to the broad bandwidth of resonance tuning. Benefiting from this broadband reconfigurable PD‐based spectroscopy, a remarkable improvement of plastic sorting accuracy was demonstrated compared to the narrowband counterparts.

## 2 Broadband Reconfigurable Photodetection and On‐Chip Spectroscopy

**Figure 1:**
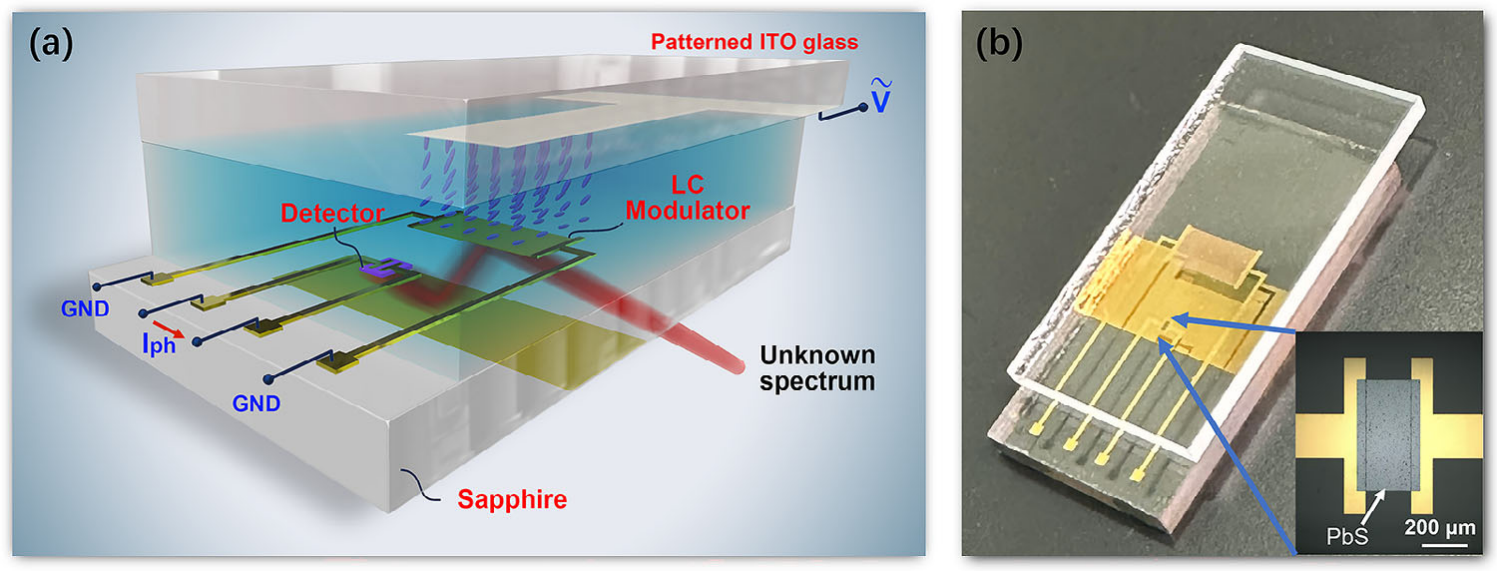
LC‐tuned reconfigurable photodetection. a) Schematic diagram and b) photograph of the proposed LC‐tuned reconfigurable PD. The prism‐coupled configuration is shown in Figure S1 (Supporting Information).

As shown in **Figure** **1**, both a light modulation unit and a photodetection unit were integrated side by side on the same piece of sapphire, where incident light is coupled to the light modulation unit via the prism coupling method^[^ ^43^ ^]^ and then the modulated light is reflected into the photodetection unit. The light modulation unit consists of a layer of LC sandwiched between a piece of Au‐coated sapphire and a piece of ITO glass. The Au film not only acts as an electrode of the LC cell but also supports SPR at the interface between Au and LC excited in a Kretschmann configuration.^[^ ^43^ ^]^ It is expected that the birefringence of LC molecules could modify the optical resonance properties and thus tune the reflection spectrum at the Au‐sapphire interface. As well known, SPR in a Kretschmann configuration has an extremely high refractive sensitivity to the dielectric environment and LC has strong birefringence. Therefore, it is expected that in such a configuration the resonance wavelength of SPR, i.e., the peak absorption wavelength, can be tuned in a broad wavelength range by changing the bias on the LC layer. On the other hand, a PbS photoconductive PD with a broad photosensitive band was fabricated directly on top of the sapphire substrate to cover the tuning wavelength range of the light modulation unit. Only the non‐resonance light can be reflected and detected by the PD. As a result, a simple reconfigurable PD can be achieved by combining the LC light modulation unit and the PbS photodetection unit. Please note that the prism does not provide any dispersion of the light here but assists light coupling, and thus no long optical path of a large prism is required. To further improve the integration, the prism can be replaced by a piece of sapphire with slanted sidewalls. With this configuration, computational spectroscopy or spectral sensing can be achieved in a compact and low‐cost platform.

### 2.1 Anisotropic Orientation of LC Molecules and Light Modulation Mechanism

As well known, LCs are made of anisotropic molecules and this anisotropy is maintained on a macroscopic scale.^[^ ^44^ ^]^ This induces strong birefringence of LCs, resulting in novel applications such as LC display. Due to high orientational order and strong birefringence, LCs usually show a high sensitivity to light polarization. To modulate light with LCs, it is important to regulate the orientation of LC molecules and thus the refractive index (RI) distribution. A unit vector *n* is usually defined to represent the average direction of the molecular long axis, which is also called the molecular director. When light is polarized along *n*, the tilt angle‐dependent effective RI of LC is the extraordinary refraction coefficient *n*_e_, in contrast, it is the ordinary refraction coefficient *n*_o_ when light is polarized in a direction perpendicular to *n*. In fact, the director field is easily distorted and aligned by magnetic and electric fields, and even by surface treatments.^[^ ^45^, ^46^ ^]^ Therefore, LCs‐based light modulation has attracted extensive research interest.

**Figure 2:**
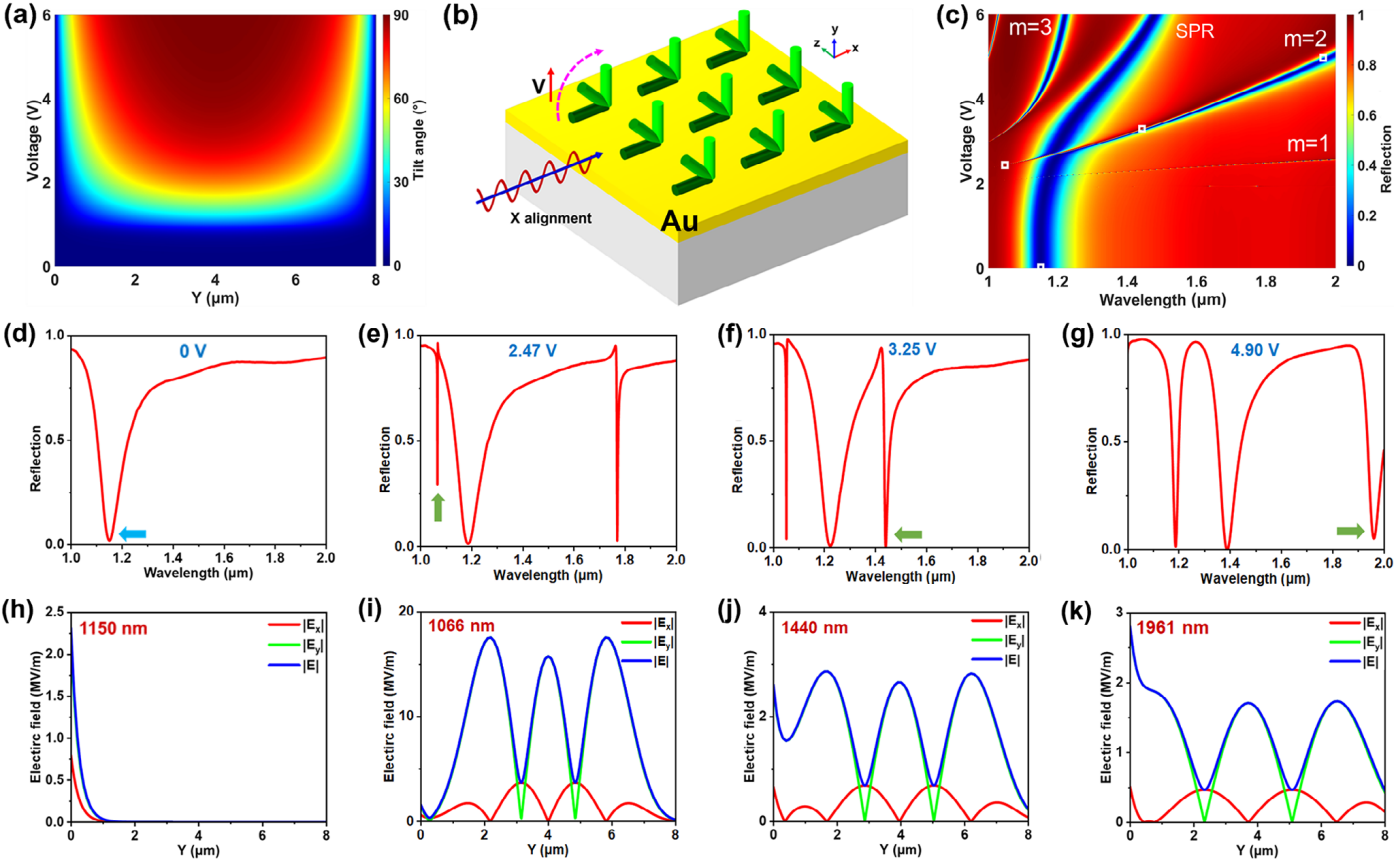
Theoretical investigation of LC molecular orientation and mode coupling in prism‐coupled LC light modulation. a) The tilted angle distribution of LC molecules at different biases assumes an infinite interface anchoring effect. The LC layer thickness *h* = 8 µm. The plane of *Y* = 0 refers to the interface between the LC layer and the SiN/Au layer. b) Schematic diagram of an LC layer pre‐aligned along *x*‐direction at the Au‐LC interface. SP wave propagates along *x*‐ direction and the electric field vector of the bias is along *y*‐ direction. c) Calculated reflectance matrix at different biases and wavelengths. d)‐g) Calculated reflection spectra at 0 V, 2.47 V, 3.25 V, and 4.90 V, respectively. h)‐k) Calculated electric field distribution at the wavelengths marked by arrows in (d)‐(g) and also marked as white squares in (c).

Lots of design works consider LC as a layer of uniform medium with a RI between *n*_e_ and *n*_o_ depending on the bias, however, the actual localized orientations of LC molecules are not uniform under bias. For example, the direction *n* of the molecules away from the interface between LCs and substrate can be easily tuned under bias but that of the molecules at the interface are hardly tuned due to the anchoring effect. It is also the main reason for the limited tuning range reported in the literatures.^[^ ^41^, ^42^ ^]^ Here, the Oseen‐Frank free energy model was used to describe the energetics of LC systems.^[^ ^47^ ^]^ In this model, the director *n* is determined by minimizing the Oseen‐Frank free energy function, which is given by

(1) $$1/2\times \int \left[K_{1}{\left(\nabla \cdot N\right)}^{2}+K_{2}{\left(N\cdot \nabla \times N\right)}^{2}+K_{3}{\left(N\times \nabla \times N\right)}^{2}\right]\mathrm{dV}$$

where *N* is the director field, and K₁, K₂, and K₃ are the splay, twist, and bend elastic constants, respectively. For the E7 LC used in this work, K₁ = 11.3 pN, K₂ = 6.7 pN, K₃ = 18.6 pN. The relative permittivity values *ε*_⊥_ = 5 and *ε*_∥_ = 18.5, represent the permittivity when the electric field is orthogonal or parallel to the director, respectively. In addition, the LC molecules at the interfaces were assumed to be anchored, i.e., the directors of all LC molecules at the interface were fixed to be aligned with the alignment layer at various biases. In this case, the orientation distribution of the LC molecules at different biases was calculated using the finite element method (FEM) based on the above model, and the results are shown in **Figure** **2**a. Below a threshold of voltage, the tilt angles of the LC molecules are close to zero no matter where they are, i.e., the LC molecules do not rotate until a threshold voltage reaches. In this case, the RI is close to *n*_o_ because the TM‐polarized surface plasmon (SP) wave has a main electric vector in a direction perpendicular to the direction *n*. Above a threshold voltage, the tilt angle increases with the increasing distance from the interface at a fixed voltage, while the tilt angles of the LC molecules closed to both interfaces are very small as expected. Complete rotation, i.e., a tilt angle of 90°, can be obtained at a large bias (e.g., 6 V for LC molecules at *Y* = 4 µm) in the center of the LC layer, where the RI is close to *n*_e_. Therefore, the LC layer sandwiched between Au and ITO glass can be seen as a gradient RI waveguide. The effective RI of LC at various voltages is shown in Figure S2 (Supporting Information). Because the RI of the LC molecule is closely connected to its orientation, it is necessary to consider such an anisotropic RI distribution in the investigation of light modulation in LC devices.

Light modulation properties in the proposed device were simulated using a model as shown in Figure 2b. Surface plasmon (SP) wave is excited at the Au‐LC interface with an oblique incident light (69.9°) via the prism coupling. During its propagation along the *x*‐direction, the SP wave interacts with LC molecules, which are pre‐aligned also along the *x*‐direction. Applying a bias between Au film and ITO glass, the director *n* of the LC molecule rotates from the *x*‐direction to the *y*‐direction, which induces a change in the localized RI of the LC layer. The reflection spectra at different biases are shown in Figure 2c. At a low voltage of less than 2 V, there is only one reflection dip, i.e., absorption peak, in a broad wavelength range of 1–2 µm, which shows negligible wavelength shift. This is associated with the threshold to rotate the LC molecules as shown in Figure 2a. Because the RI increases with the bias as shown in Figure 2a, all absorption peaks in Figure 2c show a red shift with the bias. It is interesting to know the mechanisms of this resonant absorption. At 0 V, the reflection spectrum with a main absorption peak at *λ* = 1150 nm (marked as a white square) is shown in Figure 2d. Its electric field distribution shows a typical feature of SPR with the maximum at the Au‐LC interface in Figure 2h. Increasing the bias to 6 V, the SPR wavelength increases to 1479 nm with a tuning wavelength range of 329 nm. A theoretical tuning wavelength range could further increase with a larger bias (25 V), which can be achieved of 587 nm as shown in Figure S3 (Supporting Information). Compared to the present results in the literatures, it is a remarkably high value benefiting from the extraordinary RI sensitivity of prism‐coupled SPR. Apart from the SPR, there are also waveguide modes in this gradient RI LC waveguide configuration, which may also couple to the evanescent field of total reflection as the prism‐coupled SPR and thus induce GMR‐based absorption peak in the reflection spectra. As shown in Figure 2c, multiple absorption peaks appear with a further increase of the bias. At 2.47 V, there are two additional resonances at 1066 and 1769 nm respectively as shown in Figure 2e. In contrast to the SPR, the mode field distribution of 1066 nm resonance shown in Figure 2i has the main field concentrated inside the LC layer with three antinodes, indicating a 2nd GMR. It has a much smaller linewidth than SPR due to the lower material absorption loss. The 2nd GMR shifts from 1 µm at 2.37 V to 2 µm at 5.06 V, resulting in an extraordinary wavelength tuning range of 1000 nm. Although GMR usually shows a relatively lower RI sensitivity, it is not surprising that in this structure GMR has a larger wavelength tuning range than SPR due to its concentrated mode field inside the waveguide. By integrating the sensing channel^[^ ^48^ ^]^ or the active material such as LC inside the waveguide layer, significant improvement of the RI sensitivity or light modulation efficiency can be achieved. Moreover, the tuning range can be even larger as indicated by the trend of the resonance wavelength versus the bias, which is expected to solve the issue of the limited wavelength tuning range of most on‐chip tunable spectrometers.

The fundamental and the 1st waveguide modes also induce GMRs, for example, the absorption peak at 1769 nm is associated with the 1st waveguide mode. This resonance has a high quality factor Q over 300 and thus it shows as a line in Figure 2c. The coupling of the fundamental mode is too weak to be seen in the spectra. When the bias increases to 3.25 V, the 1st GMR shifts out of the calculated wavelength range, while the SPR and the 2nd GMR shift to 1223 and 1440 nm respectively as shown in Figure 2f. The 2nd GMR still shows a concentrated field with three antinodes inside the waveguide as shown in Figure 2j. A resonance at 1052 nm appears with four antinodes inside the waveguide, which is associated with the 3rd GMR. Compared to the 2nd GMR, the 3rd GMR shows a smaller dispersion with a 220 nm tuning range from 3 to 6 V. By further increasing the bias to 4.90 V as shown in Figure 2g, the 3rd GMR, the SPR and the 2nd GMR shifts to 1188, 1389 and 1961 nm, respectively. Due to the larger resonance wavelength, the 2nd GMR shows a more extended mode field with a larger spatial overlap with the SPR mode (Figure 2k).

### 2.2 Light Modulation and Reconfigurable Photodetection

Based on the above analysis considering both the anchoring effect and the anisotropic RI distribution of LC in practical situations, it is possible to achieve a broadband light modulation. It encourages us to develop a tunable micro‐spectrometer, enabling broadband spectral sampling for integrated spectroscopic analysis. The fabrication processes of the light modulation unit and the photodetection unit are given in Figure S4 (Supporting Information). Only conventional photolithography and film deposition processes are required for the device fabrication, which greatly reduces the demand for precise nanolithography. In addition, a SiN layer was inserted between Au film and LC to tune the SP dispersion for an efficient optical coupling in a broad wavelength range (Figure S5, Supporting Information). The characterization methods of reflection spectra and photoelectric responses were given in Figure S6 (Supporting Information). The photoelectric characterization results are given in Figure S7 (Supporting Information).

**Figure 3:**
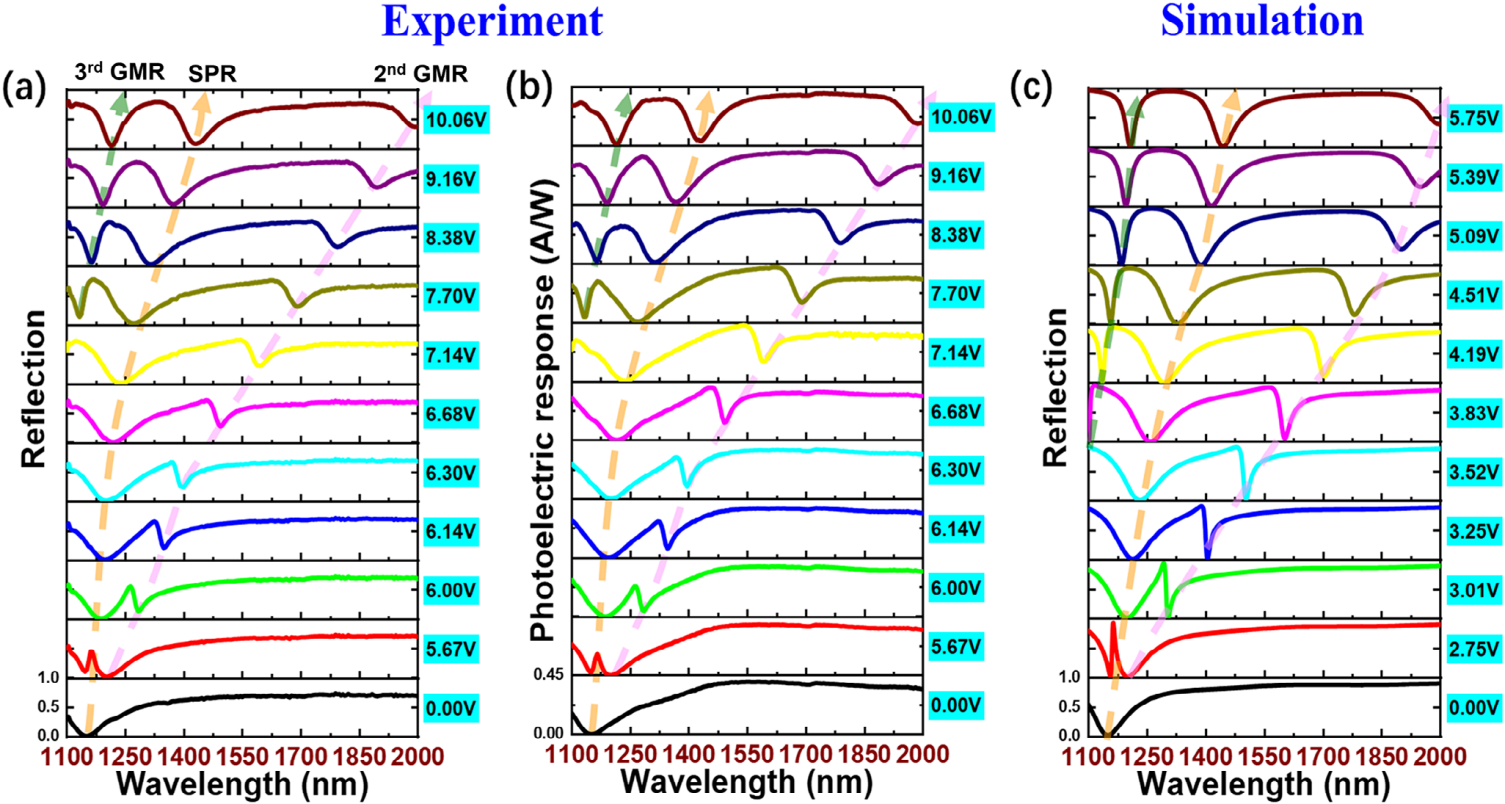
Measured spectral modulation and reconfigurable photoelectric response. a) Measured reflection spectra and b) measured photoelectric responses at different biases. The incident angle is 69.9° and the LC layer thickness is 8 µm. c) Calculated reflection spectra at different biases.

**Figure 4:**
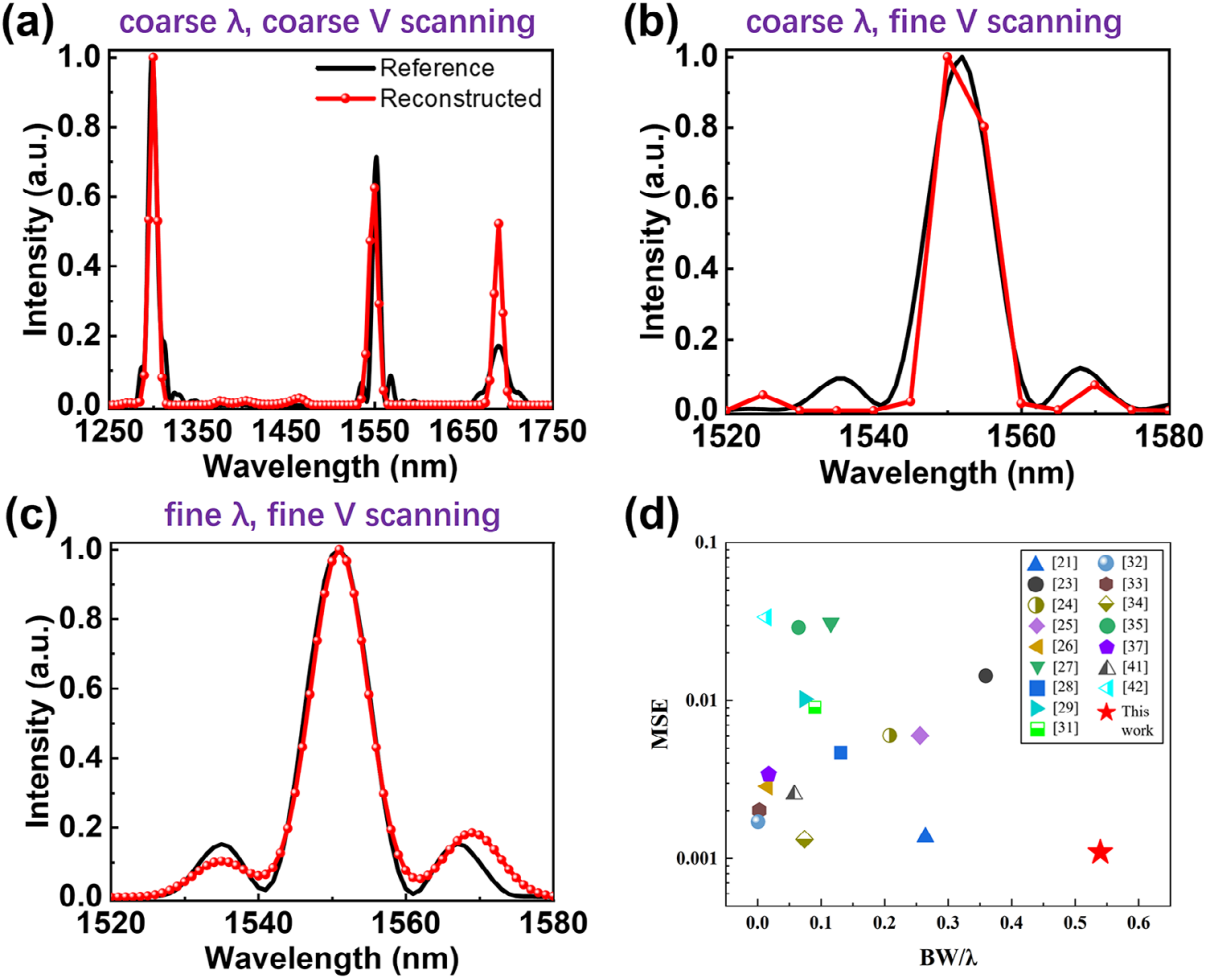
Spectral reconstruction using the proposed reconfigurable PD. Reconstructed spectra in a) 1250–1750 nm and b) 1520–1580 nm are based on the calibration and measurement with an NKT supercontinuum laser source. c) The reconstructed spectrum in 1520–1580 nm is based on the calibration and measurement with a Santec tunable laser. d) Normalized wavelength tuning bandwidths and MSEs of measured spectra with reported miniaturized spectrometers operating in a scanning mode in literature.

The measured reflection spectra at different biases with an incident angle of 69.9° are shown in **Figure** **3**a. At 0 V, there is a low reflection state at 1150 nm, which is associated with SPR‐based absorption as discussed in Figure 2. It is expected to change the RI of LC by increasing the bias, resulting in a reconfigurable reflection spectrum. No resonance shift was seen until the bias reached 5.67 V, which is in good agreement with the analysis of the threshold effect in Figure 2c. By further increasing the bias, LC molecules close to the interface start to deflect against the anchoring effect, resulting in a red shift of SPR. For a bias from 5.67 to 10.06 V, the SPR‐related reflection minimum shifts 1150 to 1428 nm, demonstrating a 278 nm tuning range in the experiment. This value is limited by the relatively low RI sensitivity of SPR of 3654 nm/RIU at 1150 nm (Figure S8, Supporting Information). When the initial resonance peak of SPR is set at 1480 nm with an incident angle of 64.9°, the tuning range over 500 nm can be achieved as shown in Figure S8 (Supporting Information) due to the higher RI sensitivity (10 043 nm/RIU) of SPR at a long wavelength. Moreover, the position dependent anchoring effect induces a gradient RI distribution of LC and thus GMR was formed in such an LC waveguide. At 5.67 V, the narrowband 2nd GMR overlaps with the broadband SPR in wavelength, where a Fano‐line‐shape transmission peak at 1160 nm was seen. As predicted in Figure 2c, GMR shifts much faster than SPR with an increasing bias. When the bias increases to 10.06 V, the GMR peak shifts to λ = 2000 nm (pink dashed line), demonstrating an extremely large wavelength tuning range over 850 nm superior to all reported results (**Figure** **4**d). Please note that this value is limited by the tuning range of the used acousto‐optic tunable filer (AOTF)polymethyl methacrylate in the experiment. The theoretical 2nd GMR peak can be tuned to over 2900 nm as shown in Figure S3 (Supporting Information). The fundamental and 1st GMR was not observed in the measured reflection spectra due to the relatively broad linewidth of the AOTF (>10 nm). In addition, the 3rd GMR was observed when the bias was over 7.7 V at a short wavelength in good agreement with Figure 2c, which also shows a redshift. The difference between the calculated voltages and the actual voltages in the experiment comes from the simplified model in our calculation. In calculation, the voltage is assumed to be applied directly across the LC layer without considering any voltage division on the SiN layer and the existing contact resistances, which are non‐negligible in the experiment and thus increase the actual voltage. Because the bias is almost proportional to the thickness of the LC layer, the voltage can be reduced if a thin LC layer is applied.^[^ ^42^ ^]^

The measured photocurrents of the PbS PD at the same 11 biases for different monochromatic illuminations are shown in Figure 3b. As expected, the variation trend of photoelectric response spectra is completely consistent with the reflection spectra in Figure 3a. The bias‐dependent evolution of three minimums in the photocurrent spectra has a similar trend with SPR, 2nd GMR, and 3rd GMR discussed in Figure 2, respectively. To verify this statement, the calculated reflection spectra at 11 different biases were plotted in Figure 3c. As seen, these 11 calculated spectra are very consistent with the 11 measured spectra. By scanning the LC bias from 0 to 10.06 V, reconfigurable photodetection with a tunable minimum photocurrent across a wavelength tuning range of 850 nm was observed in the experiment. Correspondingly, a reflection minimum associated with the 2nd GMR in simulation shifts from 1150 to 2000 nm by changing the bias from 0 to 5.92 V. To the best of our knowledge, it is the largest tunable wavelength range in NIR reported so far. Moreover, these spectra sampled at different biases have relatively low spectral correlation due to the significant difference between tunable resonances. In this case, the integrated device can be readily tuned to efficiently sample a target spectrum by scanning the bias, which contributes to spectral sensing and spectroscopy. Benefiting from both the unique broadband light modulation scheme and broadband PbS PD, a scanning integrated spectroscopy can be achieved in a broadband.

### 2.3 Reconfigurable Spectral Reconstruction

Reconfigurable photodetectors provide a spectral sampling method operating in a scanning mode. For spectral reconstruction, a response matrix is usually calibrated in advance,^[^ ^13^ ^]^ which consists of photocurrents under different monochromatic illumination at different biases. The details of calibration and spectral reconstruction are provided in Figure S9 (Supporting Information). For a target spectrum, the device photocurrents under this illumination should be recorded at different biases, and then together with the response matrix are used in reconstruction via the compressive sensing (CS) algorithm^[^ ^49^ ^]^ or DL algorithm.^[^ ^50^ ^]^

First, the device calibration was done using an NKT supercontinuum laser source together with an AOTF (linewidth 6.4‐19.8 nm for λ = 1100–2000 nm) at a wavelength step of 5 nm, where in total 21 voltages were used in a scanning mode to sample the spectral response in a wavelength range of 1250–1750 nm. A customized illumination using the NKT laser source was used in the spectral reconstruction experiment as shown in Figure 4a, where the ground truth was obtained using a commercial spectrometer (Anritsu MS9740A). The reconstructed spectrum in 1250–1750 nm can accurately locate the peaks with a deviation of the peak wavelengths of 1–2 nm. Considering such an undersampling situation (21 samplings in 500 nm), the CS algorithm works well. However, some small peaks are missing, for example, the two sidelobes around the main peak at 1550 nm. Then, the bias scanning at 9 voltages for the same illumination was redone in a range of 1520–1580 nm at the same wavelength step of 5 nm. Interestingly, the missing sidelobes appear as shown in Figure 4b due to the improved spectral sampling (9 samplings in 60 nm), but the resolution is still limited by the linewidth of the AOTF (≈10 nm). Because the spectral sampling accuracy is associated with the linewidth of the laser source, finer calibration and measurement were done using a Santec tunable laser (linewidth 200 kHz) at a step of 1 nm in 1520–1580 nm under in total 9 voltages. As shown in Figure 4c, both peak wavelengths and spectral profiles are in good agreement with the ground truths. Due to the reconfigurable advantage of such an LC‐based single‐pixel spectrometer, coarse scanning and fine scanning can be combined in practical applications to save measurement time and simultaneously obtain accurate spectral information in an interesting wavelength range. In addition, the result of the spectral reconstruction of a broadband spectrum (transmission through a piece of polymethyl methacrylate, PMMA) was demonstrated in Figure S9 (Supporting Information), where the reconstructed spectrum agrees with the result of a commercial spectrometer reasonably well. The relatively large deviation comes from the change of the measurement setups, i.e., the AOTF was disconnected from the NKT light source and removed from the light path because the AOTF has only eight narrowband output channels. Figure 4d shows normalized wavelength tuning bandwidths and mean square errors (MSEs) of measured spectra with reported miniaturized spectrometers operating in a scanning mode in literature.

## 3 High‐Accuracy Plastic Sorting via Broadband Spectral Sensing

**Figure 5:**
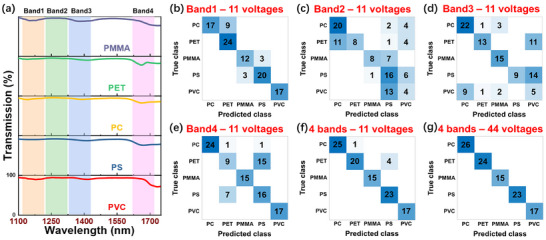
Spectral sensing via reconfigurable photodetection. a) Measured transmission spectra of PMMA, PET, PC, PS, and PVC in a thickness of 1 mm by a commercial spectrometer. Four bands were indicated by colored regions, where Band 1 covers 1118–1218 nm, Band 2 covers 1224–1324 nm, Band 3 covers 1330–1430 nm and Band 4 covers 1620–1720 nm. b)‐g) Plastics sorting results of 105 plastic samples using the reconfigurable PD under different scanning voltages. All voltages are listed in Table S2 (Supporting Information).

Such a spectral sampling method in a scanning mode can also be used for spectral sensing in material analysis. In this case, the complete spectrum information is not necessary. Instead, the photoelectric responses of the device at different biases were recorded for the analyte under different monochromatic illumination and used for training. Here, five types of plastics including polystyrene (PS), polyvinyl chloride (PVC), polyethylene terephthalate (PET), polycarbonate (PC), and PMMA were chosen to conduct the plastic sorting experiment using the proposed device. Although all these five materials are transparent in visible, they show some differences in NIR transmission spectra measured by a commercial spectrometer as shown in **Figure** **5**a. In total 420 different samples were used to collect the photoelectric responses for training. As shown in Figures S10a,d (Supporting Information), the photoelectric response spectra of the reconfigurable PD at 44 voltages (6.1–17.5 V) were plotted under the broadband illumination of the NKT supercontinuum laser source (without an AOTF). For the 1st (6.1–8.1 V), 2nd (8.2–9.2V ), 3rd (9.4–10.4 V) and 4th (13.5–17.5 V) 11‐voltage set, the SPR appears in Band 1 (1118–1218 nm, yellow region in Figure 5a), Band 2 (1224–1324 nm, green region in Figure 5a), Band 3 (1330–1430 nm, blue region in Figure 5a) and Band 4 (1620–1720 nm, pink region in Figure 5a) respectively, which dominates the main photocurrent modulation. The transmitted light through different plastic samples under the same illumination was recorded by the reconfigurable PD at the same 44 voltages as shown in Figures S10e,h (Supporting Information). These output photocurrents contain spectral absorption related features of various plastics, which were used for spectral sensing with a linear discriminant algorithm^[^ ^51^ ^]^ (Figure S10, Supporting Information). With the photocurrent data in Band 1 to Band 4, the average plastics sorting accuracy is 85.71%, 53.33%, 60.95%, and 77.14% respectively as shown in Figure 5b–e. In all cases, the plastic sorting accuracy is limited by the insufficient sampling of spectral information in a narrow band. Benefiting from the broadband advantage of the proposed reconfigurable PD, it is possible to conduct the plastics sorting in all four bands. As shown in Figure 5f, the sorting accuracy significantly increases to 95% although the number of the sampling voltages is also 11. By further increasing the number of the sampling voltages to 44, 100% plastic sorting accuracy was achieved as shown in Figure 5g. These results show the advantage of the broadband reconfigurable PD for spectral sensing. Moreover, the proposed device provides high flexibility for different analytes by simply setting the voltages in a scanning operation.

## 4 Conclusion

In this work, we demonstrated an NIR miniaturized spectrometer operating in a scanning mode based on a single reconfigurable PD. The wavelength tuning range reaches a remarkable value of over 850 nm, which is the highest reported result to the best of our knowledge. Considering the actual anisotropic RI distribution and the interface anchoring effect of LC, the complex light modulation behavior was revealed by FEM simulation, which is in good agreement with the measured results. It is indicated that the great dispersion of prism‐coupled SPR and GMR contributes to the large wavelength tuning range. By sampling the spectral information in a scanning mode via tuning the LC bias, the reconstruction deviation at a peak wavelength of 1550 nm reaches 1 nm. Remarkable improvement in plastic sorting accuracy was demonstrated benefiting from this broadband reconfigurable PD based spectroscopy in comparison with the narrowband counterpart. Moreover, only conventional photolithography and film deposition processes are required for device fabrication, which greatly reduces the demand for precise nanolithography. Furthermore, it can be further miniaturized down to a mm^3^ scale by combining the detection unit and the modulation unit into one device structure. For example, an Au/Si hot electron detection unit instead of a PbS PD can be vertically integrated with the LC modulation unit, where the Au layer is used as electrodes of both units. In such a configuration, the absorbed light in Au contributes to the hot electron induced photocurrent, simultaneously, the spectrum of the absorbed light is manipulated via the tuned SPR by changing the LC bias. In comparison to snapshot micro‐spectrometers, the proposed device based on a reconfigurable PD has a lower cost, and a higher signal‐to‐noise ratio due to a larger PD, and can be post‐tuned after manufacturing for different application scenarios. It is suitable for scenarios that do not require high measuring speed but are sensitive to integration and cost.

## Conflict of Interest

The authors declare no conflict of interest.

## Author Contributions

W.L, X.N. and W.C. equally contributed to this work. L.W. and Q.C. conceived the idea and designed the experiments. W.L., L.W., J. Y., and D.L. performed the simulation. X.N., W.C., and Y.H. fabricated the devices. W.L., N.T., and Q.Z. did the optical/electrical measurements. W.L. and Y.L. performed the spectra reconstruction. Q.C., L.W., and Y.L. supervised the project. W.L. and Q.C. analyzed the results and prepared the manuscript. All authors contributed to the scientific discussion.

## Supporting information



Supporting Information

## Data Availability

The data that support the findings of this study are available from the corresponding author upon reasonable request.;

## References

[1] N. V. Tkachenko , Optical Spectroscopy: Methods and Instrumentations, Elsevier, Amsterdam 2006.

[2] Z. Yang , T. Albrow‐Owen , W. Cai , T. Hasan , Science 2021, 371, 480. 10.1126/science.abe0722 33509998

[3] A. Li , C. Yao , J. Xia , H. Wang , Q. Cheng , R. Penty , Y. Fainman , S. Pan , Light: Sci. Appl. 2022, 11, 174. 35672298 10.1038/s41377-022-00853-1 PMC9174208

[4] Q. Chen , L. Liang , Q. Zheng , Y. Zhang , L. Wen , Opto‐Electron. Adv. 2020, 3, 190040.

[5] S. Grabarnik , R. Wolffenbuttel , A. Emadi , M. Loktev , E. Sokolova , G. Vdovin , Opt. Express 2007, 15, 3581. 19532602 10.1364/oe.15.003581

[6] S. Johann , M. Mansurova , H. Kohlhoff , A. Gkertsos , P. P. Neumann , J. Bell , M. Bartholmai , IEEE Sensors Proc. 2018, 1067.

[7] S. W. Wang , C. Xia , X. Chen , W. Lu , Opt. Lett. 2007, 32, 632. 17308584 10.1364/ol.32.000632

[8] J. Bao , M. G. Bawendi , Nature 2015, 523, 67. 26135449 10.1038/nature14576

[9] Z. Wang , S. Yi , A. Chen , M. Zhou , T. S. Luk , A. James , J. Nogan , W. Ross , G. Joe , A. Shahsafi , K. X. Wang , M. A. Kats , Z. Yu , Nat. Commun. 2019, 10, 1020. 30833569 10.1038/s41467-019-08994-5 PMC6399238

[10] J. Xiong , X. Cai , K. Cui , Y. Huang , J. Yang , H. Zhu , W. Li , B. Hong , S. Rao , Z. Zheng , S. Xu , Y. He , F. Liu , X. Feng , W. Zhang , Optica 2022, 9, 461.

[11] D. Tua , R. Liu , W. Yang , L. Zhou , H. Song , L. Ying , Q. Gan , Nat. Commun. 2023, 14, 1902. 37019920 10.1038/s41467-023-37628-0 PMC10076426

[12] Q. L. Zheng , X. Nan , B. Chen , H. Wang , H. Nie , M. Gao , Z. Liu , L. Wen , D. R. S. Cumming , Q. Chen , Laser Photon. Rev. 2023, 17, 2300475.

[13] Z. Yang , T. Albrow‐Owen , H. Cui , J. Alexander‐Webber , F. Gu , X. Wang , T. Wu , M. Zhuge , C. Williams , P. Wang , A. V. Zayats , W. Cai , L. Dai , S. Hofmann , M. Overend , L. Tong , Q. Yang , Z. Sun , T. Hasan , Science 2019, 365, 1017. 31488686 10.1126/science.aax8814

[14] L. Gao , Y. R. Qu , L. H. Wang , Z. F. Yu , Nanophotonics 2022, 11, 2507. 39635673 10.1515/nanoph-2021-0636 PMC11502016

[15] C. Chen , H. Gu , S. Liu , Light: Sci. Appl. 2024, 13, 9. 38177112 10.1038/s41377-023-01355-4 PMC10766968

[16] L. Wen , Q. Chen , X. Hu , H. Wang , L. Jin , Q. Su , ACS Nano 2016, 10, 11076. 28024346 10.1021/acsnano.6b05960

[17] Q. Chen , X. Nan , M. Chen , D. Pan , X. Yang , L. Wen , Adv. Mater. 2021, 33, 2103815. 10.1002/adma.202103815 34595789

[18] X. Nan , W. Lai , J. Peng , H. Wang , B. Chen , H. He , Z. Mo , Z. Xia , N. Tan , Z. Liu , L. Wen , D. Gao , Q. Chen , Adv. Photonics 2024, 6, 026007.

[19] L. Wen , Z. Sun , Q. Zheng , X. Nan , Z. Lou , Z. Liu , D. R. S. Cumming , B. Li , Q. Chen , Light: Sci. Appl. 2023, 12, 76. 36944614 10.1038/s41377-023-01123-4 PMC10030554

[20] L. Wen , L. Li , X. Yang , Z. Liu , B. Li , Q. Chen , ACS Nano 2019, 13, 6963. 31180202 10.1021/acsnano.9b01914

[21] H. H. Yoon , H. A. Fernandez , F. Nigmatulin , W. Cai , Z. Yang , H. Cui , F. Ahmed , X. Cui , Md. G. Uddin , E. D. Minot , H. Lipsanen , K. Kim , P. Hakonen , T. Hasan , Z. Sun , Science 2022, 378, 296. 36264793 10.1126/science.add8544

[22] L. Guo , H. Sun , M. Wang , M. Wang , L. Min , F. Cao , W. Tian , L. Li , Adv. Mater. 2022, 34, 2200221. 10.1002/adma.202200221 35706366

[23] W. Deng , Z. Zheng , J. Li , R. Zhou , X. Chen , D. Zhang , Y. Lu , C. Wang , C. You , S. Li , L. Sun , Y. Wu , X. Li , B. An , Z. Liu , Q. J. Wang , X. Duan , Y. Zhang , Nat. Commun. 2022, 13, 4627. 35941126 10.1038/s41467-022-32306-z PMC9360404

[24] Z. Zhou , Y. Zhang , Y. Xie , T. Huang , Z. Li , P. Chen , Y. Lu , S. Yu , S. Zhang , G. Zheng , Light: Sci. Appl. 2024, 13, 242. 39245765 10.1038/s41377-024-01608-w PMC11381520

[25] J. Wang , B. Pan , Z. Wang , J. Zhang , Z. Zhou , L. Yao , Y. Wu , W. Ren , J. Wang , H. Ji , J. Yu , B. Chen , Nat. Commun. 2024, 15, 1773. 38413622 10.1038/s41467-024-46066-5 PMC10899627

[26] M. Tian , B. Liu , Z. Lu , Y. Wang , Z. Zheng , J. Song , X. Zhong , F. Wang , Light: Sci. Appl. 2024, 13, 278. 39341832 10.1038/s41377-024-01638-4 PMC11438984

[27] X. Du , Y. Wang , Y. Cui , G. Rao , J. Huang , X. Chen , T. Zhou , C. Wu , Z. Yang , H. Cui , Y. Zhao , J. Xiong , Nat. Electron. 2024, 7, 984.

[28] Y. August , A. Stern , Opt. Lett. 2013, 38, 4996. 24281493 10.1364/OL.38.004996

[29] D. M. Kita , B. Miranda , D. Favela , D. Bono , J. Michon , H. Lin , T. Gu , J. Hu , Nat. Commun. 2018, 9, 4405. 30353014 10.1038/s41467-018-06773-2 PMC6199339

[30] L. Liang , Q. Zheng , L. Wen , D. Cumming , Q. Chen , Opt. Lett. 2021, 46, 4264. 34469990 10.1364/OL.426624

[31] Q. Qiao , X. Liu , Z. Ren , B. Dong , J. Xia , H. Sun , C. Lee , G. Zhou , ACS Photonics 2022, 9, 2367.

[32] L. Zhang , M. Zhang , T. Chen , D. Liu , S. Hong , D. Dai , Opto‐Electron. Adv. 2022, 5, 210100.

[33] H. Xu , Y. Qin , G. Hu , H. K. Tsang , Light: Sci. Appl.‐ 2023, 12, 64. 36872369 10.1038/s41377-023-01102-9 PMC9986235

[34] C. Yao , M. Chen , T. Yan , L. Ming , Q. Cheng , R. Penty , Light: Sci. Appl. 2023, 12, 156. 37357227 10.1038/s41377-023-01195-2 PMC10290986

[35] H. Chen , H. Zhang , J. Zhou , C. Ma , Q. Huang , H. Wang , Q. Ren , N. Wang , C. Lee , Y. Ma , Photonics Res. 2024, 12, 1730.

[36] L. Z. Zhang , Q. Cao , Y. Zuo , C. Xue , B. Cheng , Q. Wang , IEEE Photonics Technol. Lett. 2011, 23, 881.

[37] S. N. Zheng , J. Zou , H. Cai , J. Song , L. Chin , P. Liu , Z. Lin , D. Kwong , A. Liu , Nat. Commun. 2019, 10, 2349. 31138800 10.1038/s41467-019-10282-1 PMC6538731

[38] W. J. Yang , A. Gerke , L. Zhu , C. Chase , Y. Rao , J. Chang‐Hansnain , IEEE J. Sel. Top. Quantum Electron. 2014, 20, 178.

[39] H. Mao , K. Silva , M. Martyniuk , J. Antoszewski , J. Bumgarner , B. Nener , J. Dell , L. Faraone , J. Microelectromech. Syst. 2016, 25, 227.

[40] L. Wen , X. Nan , J. Li , R. S. C. Cumming , X. Hu , Q. Chen , Opto‐Electron. Adv. 2022, 5, 200093.

[41] C. Levallois , B. Sadani , B. Boisnard , T. Camps , C. Paranthoen , S. Pes , S. Bouchoule , L. Dupont , J. Doucet , M. Alouini , B. Bardinal , Opt. Express 2018, 26, 25952. 30469689 10.1364/OE.26.025952

[42] Y. Ni , C. Chen , S. Wen , X. Xue , L. Sun , Y. Yang , eLight 2022, 2, 23.

[43] J. Homola , S. S. Yee , G. Gauglitz , Sens. Actuat. B:Chem. 1999, 54, 3.

[44] F. Simoni , O. Francescangeli , J. Phys.: Condens. Matter 1999, 11, R439.

[45] G. Babakhanova , O. D. Lavrentovich , The Techniques of Surface Alignment of Liquid Crystals, Springer International Publishing, Kyiv, Ukraine 2019.

[46] D. Kang , H. Heo , Y. Yang , J. Seong , H. Kim , J. Kim , J. Rho , Opto‐Electron. Adv. 2024, 7, 230216.

[47] M. Kleman , O. D. Lavrentovich , Soft Matter Physics: An Introduction, Springer, New York 2003.

[48] L. Liang , X. Hu , L. Wen , Y. Zhu , X. Yang , J. Zhou , Y. Zhang , I. Carranza , J. Grant , C. Jiang , R. S. C. Cumming , B. Li , Q. Chen , Laser Photon Rev. 2018, 12, 1800078.

[49] G. Wu , M. Abid , M. Zerara , J. Cho , M. Choi , C. Coileain , K. Hung , C. Chang , I. Shvets , H. Wu , Nat. Commun. 2024, 15, 676. 38263315 10.1038/s41467-024-44884-1 PMC10805890

[50] D. Tua , R. Liu , W. Yang , L. Zhou , H. Song , Q. Gan , Nat. Commun. 2023, 14, 1902. 37019920 10.1038/s41467-023-37628-0 PMC10076426

[51] A. Tharwat , T. Gaber , A. Ibrahim , A. E. Hassanien , Ai Commun 2017, 30, 169.

