# Supplementary material for: Single‐Pixel Infrared Miniaturized Spectrometer Enabled by Ultra‐Broadband Reconfigurable Photodetection
Source: Adv Sci (Weinh). 2025 Apr 2;12(25):2500830. doi: 10.1002/advs.202500830 (PMC12224978; doi:10.1002/advs.202500830)
Supplement: Supplementary file 1 — Supporting Information [file ADVS-12-2500830-s001.docx]

**Supplementary**

**Single-Pixel Infrared** **Miniaturized Spectrometer Enabled**

**by Ultra-Broadband Reconfigurable Photodetection**

*Wenyue Liang^1,#^, Xianghong Nan^1,#^, Wenfeng Cai^2,#^, Ning Tan^1^, Qilin Zheng^1^, Yuyao Lu^1^, Yongyue Huang^1^, Jiahao Yan^1^, Dangyuan Lei^3^, Long Wen^1,*^, Yanjun Liu^2,*^, Qin Chen^1,*^*

^1^Guangdong Provincial Key Laboratory of Nanophotonic Manipulation, Institute of Nanophotonics, College of Physics & Optoelectronic Engineering, Jinan University, Guangzhou 511443, China

^2^Department of Electrical and Electronic Engineering, Southern University of Science and Technology, Shenzhen 518055, China

^3^Department of Materials Science and Engineering, City University of Hong Kong, Kowloon, Hong Kong 999077, China

^#^equal contribution

[*longwen@jnu.edu.cn](mailto:*longwen@jnu.edu.cn), *[yjliu@sustech.edu.cn](mailto:yjliu@sustech.edu.cn), [*chenqin2018@jnu.edu.cn](mailto:*chenqin2018@jnu.edu.cn)

Table S1**.** Wavelength tuning range of reconfigurable photodetectors in literatures

Table S2. 44 biases in the plastic sorting experiment

S1**.** Schematics of prism-coupled configuration

S2. The effective refractive index of liquid crystal

S3. Theoretical wavelength tuning ranges

S4. Fabrication processes

S5. SP dispersion tuning

S6. Optical and photoelectric characterization methods

S7**.** Photoelectric characterization of the PbS photodetector

S8. Wavelength-dependent refractive index sensitivity

S9**.** Spectral reconstruction

S10. Plastic sorting

**Table S1.** Wavelength tuning range of reconfigurable photodetectors in literatures

| **Num** | **Mechanism** | **Spectral tuning range** | **Resolution** | **Response time** | **Ref** |
| --- | --- | --- | --- | --- | --- |
| 1 | 2D heterostructure | 575-750 nm | 3 nm | / | [21] |
| 2 | 2D heterostructure | 1078-1550 nm | 20 nm | 20 ms | [23] |
| 3 | LC | 560-690 nm | / | 100 ms | [24] |
| 4 | Homojunction | 580-750 nm | 10 nm | / | [25] |
| 5 | Electrochromic | 635-645 nm | 0.29 nm | 32 ms | [26] |
| 6 | 2D homojunction | 678-761 nm | 1.2 nm | 200 ms | [27] |
| 7 | Liquid crystal | 500-570 nm | 0.4 nm | / | [28] |
| 8 | Fourier transform | 1501-1616 nm | 0.2 nm | 2.7 s | [29] |
| 9 | MEMS | 3700-4050 nm | 3 nm | 11.5 μs | [31] |
| 10 | Cascade ring | 1546-1547 nm | 5 pm | / | [32] |
| 11 | Micro-ring resonators | 1500-1504 nm | 40 pm | / | [33] |
| 12 | Micro-ring resonators | 1501-1616 nm | 30 pm | 0.3 s | [34] |
| 13 | MEMS | 1500-1600 nm | 0.2 nm | / | [35] |
| 14 | Micro-ring resonators | 1588-1616 nm | 0.47 nm | 0.2 s | [37] |
| 15 | LC+FP | 1510-1600 nm | / | / | [41] |
| 16 | LC+metasurface | 1430-1450 nm | 3 nm | 6 ms | [42] |
| 17 | **LC+SPR/GMR** | **1150-2000nm** | **2 nm** | **100 ms** | **This work** |

Note: The reference Nos in the table are the same as those in the main text.

LC: liquid crystal, FP: Fabry Perot, SPR: surface plasmon resonance, GMR: guided mode resonance

1. **Schematics of prism-coupled configuration**

Figure S1 shows the prism-coupled configuration for LC tuned photodetection. Both a light modulation unit (LC/Au) and a photodetection unit (PbS) are integrated side by side on a same piece of sapphire. Except the on-resonance incident light absorbed by the Au layer, the rest of incident light can be reflected to the PbS photodetector. Thus, the spectrum of the detected light is reconfigurable by tuning the bias on the LC layer.


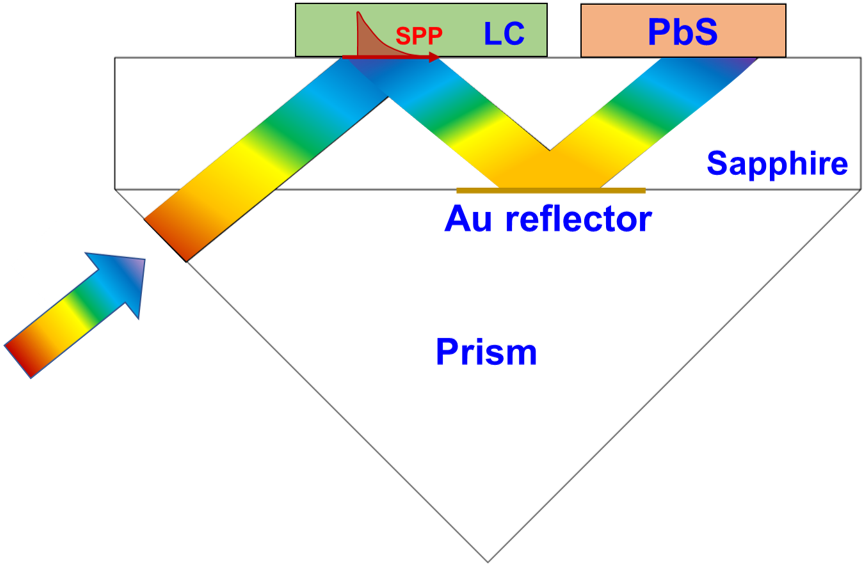


Figure S1. Schematic diagram of prism-coupled configuration for LC tuned photodetection.

1. **The effective refractive index of LC**

The effective refractive index *n*_eff_ is derived from the orientation-dependent birefringence of LC. When the molecules deflect by an angle *θ*, *n*_eff_ can be expressed as:

$n_{\text{eff}}=\frac{n_{\text{e}}n_{\text{o}}}{\sqrt{\left( n_{\text{e}}\cos\theta\right)^{2}+\left( n_{\text{o}}\sin\theta\right)^{2}}}$ (equ. S1)

where *n*_o_ and *n*_e_ are the ordinary and extraordinary refractive indices of the LC, respectively. Figure S2 shows the distribution of effective refractive index of the LC layer under different voltages. At 0 V, an effective refractive index of n_o_ (~1.5) is observed across the entire thickness of the LC layer. The effective refractive index increases with the increasing bias, where the central part of the LC layer in the *Y* direction first reaches the maximum of *n*_e_ (~1.7) associated with a maximum deflection angle (90º) of the LC molecules. In contrast, the effective refractive index remains *n*_o_ due to the surface anchoring effect. Such an effective refractive index distribution leads to a waveguide structure.


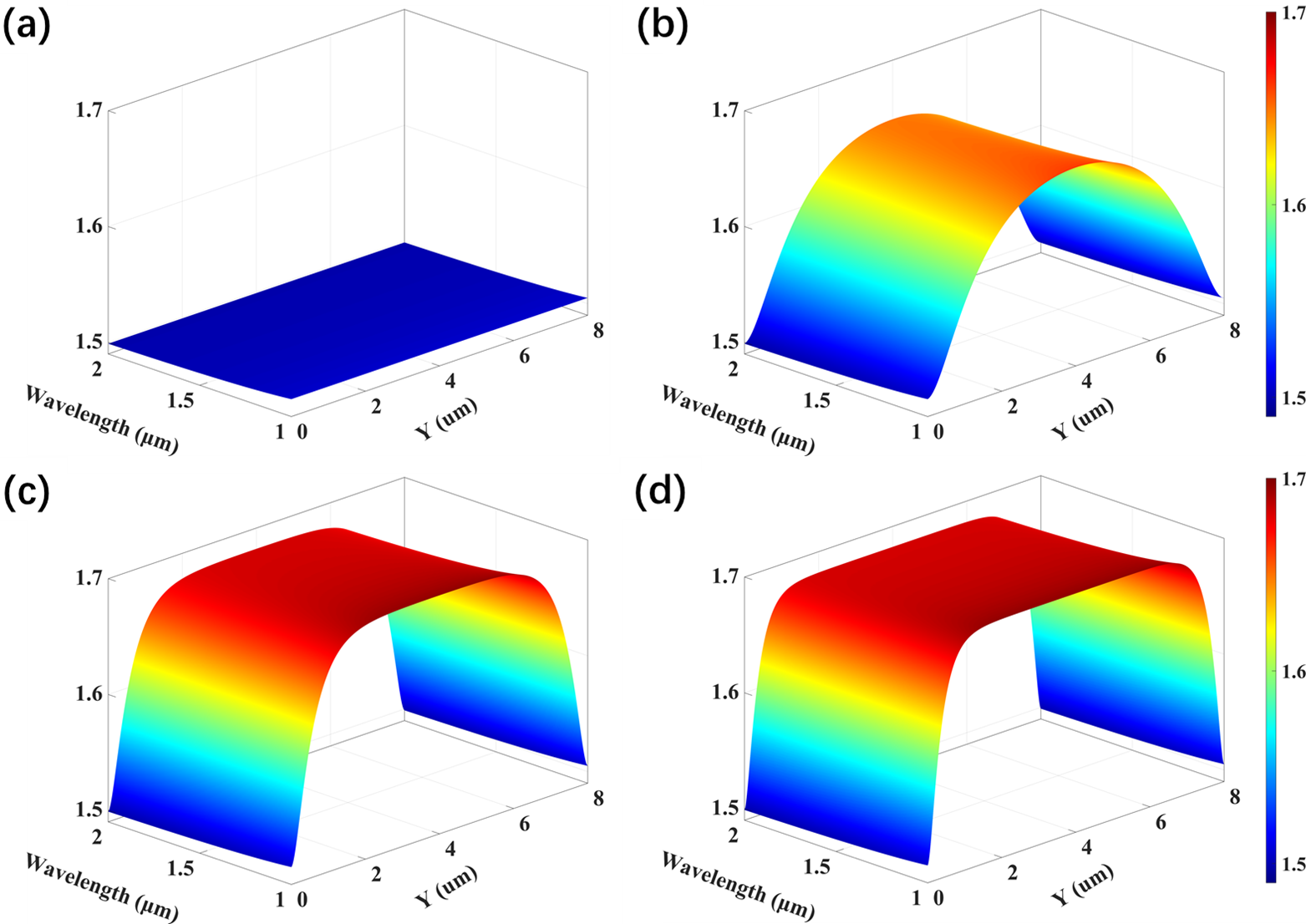


Figure S2. Effective refractive index of LC at (a) 0 V, (b) 2 V, (c) 4 V and (d) 6 V, respectively.

1. **Theoretical wavelength** **tuning ranges**

It is interesting to know the potential wavelength tuning range of various resonances shown in Figure 2c. By increasing the bias up to 25 V in simulation, the calculated reflectance matrix at different biases and wavelengths is shown in Figure S3. The 2^nd^ GMR peak shows a red shift from 1000 nm to 2926 nm with a tuning range above 1900 nm. Such a 100% (bandwidth/λ_0_) relative tuning range is the highest reported result to the best of our knowledge. In contrast, SPR suffers from the surface anchoring effect of LC molecules and exhibits a tuning range of 587 nm from 1150 nm to 1737 nm. The 1^st^ GMR is too weak to be used for spectral sampling, and other higher order resonances have limited wavelength tuning ranges.


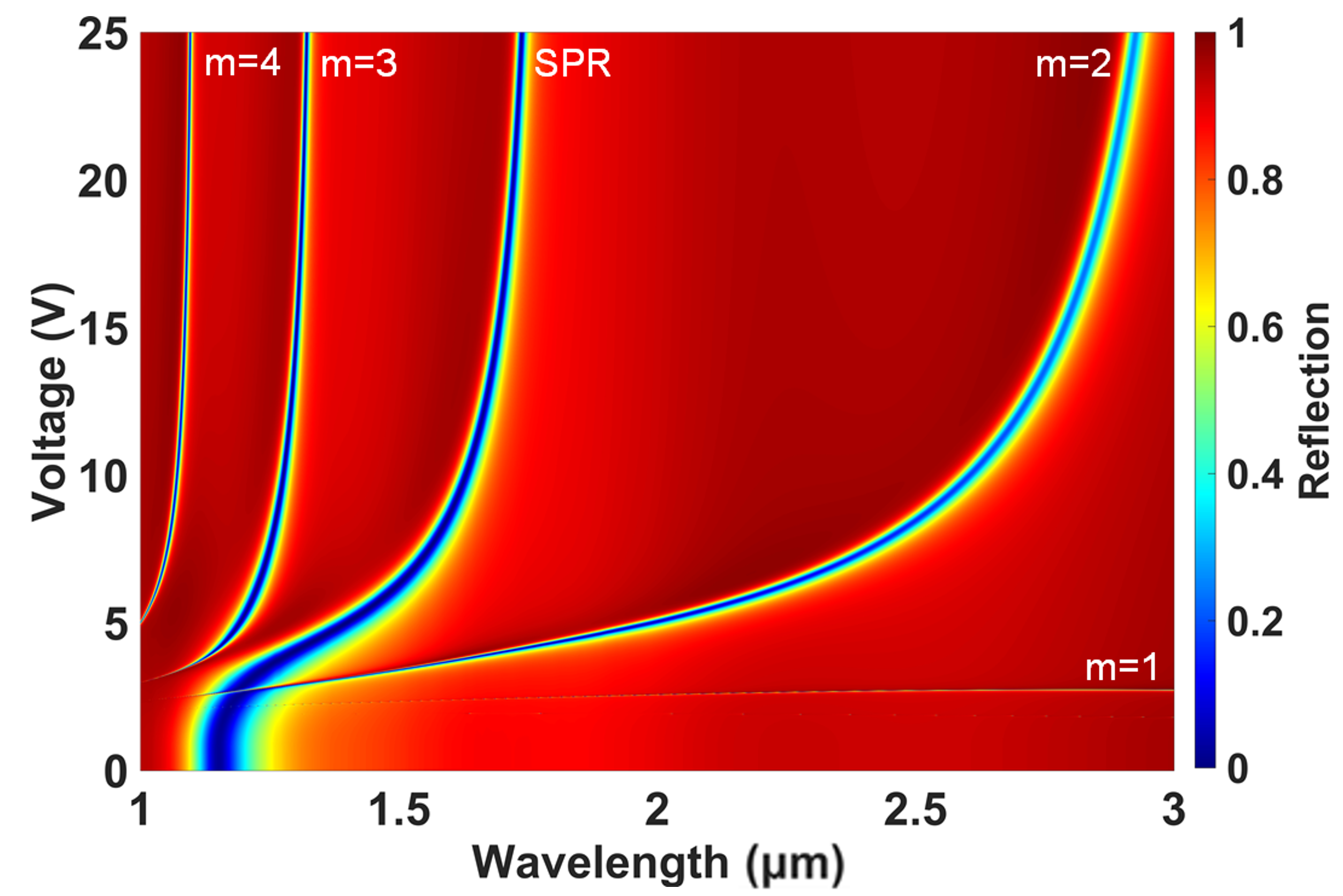


**Figure S3.** Calculated reflectance matrix at different biases and wavelengths.

1. **Fabrication processes**

Both photodetection unit and light modulation unit were fabricated on a same piece of sapphire substrate as shown in Figure 1. The photodetection unit was fabricated first.

**PbS photodetection unit**

A chemical bath deposition (CBD) method was used to synthesize PbS polycrystalline photosensitive film. First, Pb(NO₃)₂, NaOH, and SC(NH₂)₂ in a molar ratio of 3:1:1 were dissolved in deionized water. The solution was stirred using a magnetic stirrer, and the film deposition on a sapphire substrate was carried out at a water bath temperature of 30°C. The thickness of the as-deposited PbS film is approximately 500 nm. Then, the sample was sensitized at 500°C for 60 minutes in an air atmosphere using a resistance furnace to improve the <200> crystal orientation and facilitate the incorporation of oxygen at the PbS grain boundaries, thereby improving photosensitivity. A PbS photoconductive detector with a channel length of 200 μm was finally fabricated by photolithography and magnetron sputtering. Finally, the active region was coated with a layer of Al_2_O_3_ by atomic layer deposition for protection.

**LC light modulation unit**

The process started with photolithography and deposition of a 40 nm Au layer and a 50 nm SiN layer on a same piece of sapphire substrate with the photodetection unit to define the region of light modulation unit. Then, SD1 was coated for LC pre-alignment. A piece of ITO glass coated with PI for pre-alignment was placed face down to the sapphire substrate to form a LC box with a cell gap of approximately 8 μm, which was obtained by inserting glass spacers at the corners of the cell. Finally, nematic E7 LC filled the gap by capillary action.

1. **SP dispersion tuning**

The detailed structure of the LC light modulation unit is shown in Figure S4. A N-SF11 prism (*n* = 1.75) is attached to the bottom surface of the sapphire substrate. A thin layer of Au (40 nm) is used to support SPR via prism coupling and acts an electrode to add a bias to the LC layer. A SiN layer of 50 nm is inserted between Au and LC to tune the SP dispersion. As shown in Figure S5a, the dispersion of the SP wave shows a monotonic function between the incident angle and the wavelength of SPR. By changing the incident angle, SPR at any wavelength between 1-2 μm can be excited. For example, SPR resonances at 1100 nm, 1496 nm and 1866 nm can be excited at an incident angle of 70.5°, 65.2° and 63.7° respectively as marked by 1, 2 and 3 in Figure S5a. In contrast, the resonance is wider for a same stack without SiN as shown in Figure S5b. Moreover, the dispersion is flattened for an incident angle larger than 61.7°, i.e., no distinct absorption peak (reflection dip) at a wavelength larger than 1.5 μm can be achieved. For example, a broad reflection dip covering 1400-2000 nm can be observed at an incident angle of 61.6°. All these are unfavorable for achieving accurate spectral information sampling. Therefore, in experiment a SiN layer was added between Au and LC. Such a thin layer of 50 nm has negligible influence on the optical field distribution, which extends perpendicularly over a micron scale.


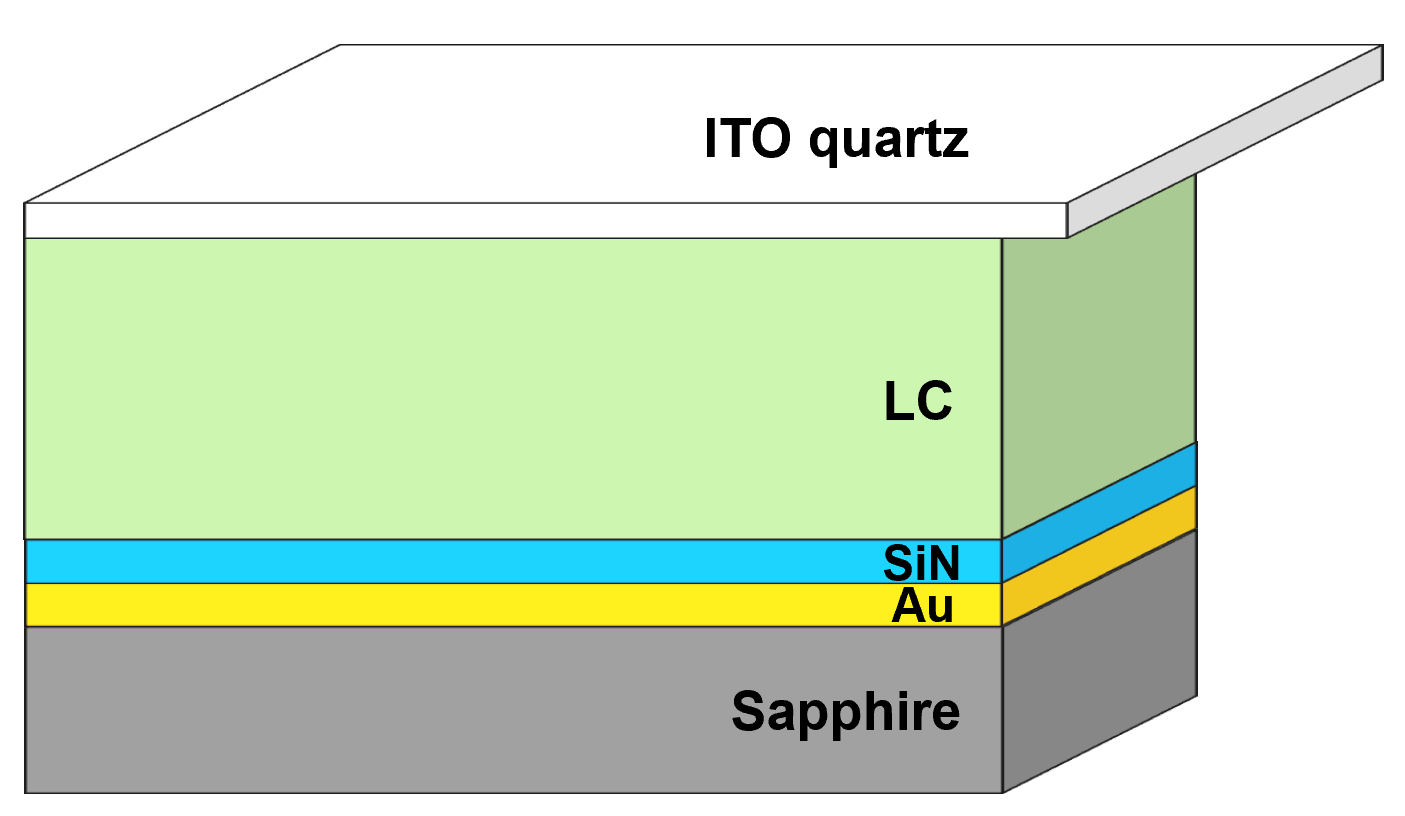


**Figure S4.** Schematic diagram of the LC light modulation unit.

**
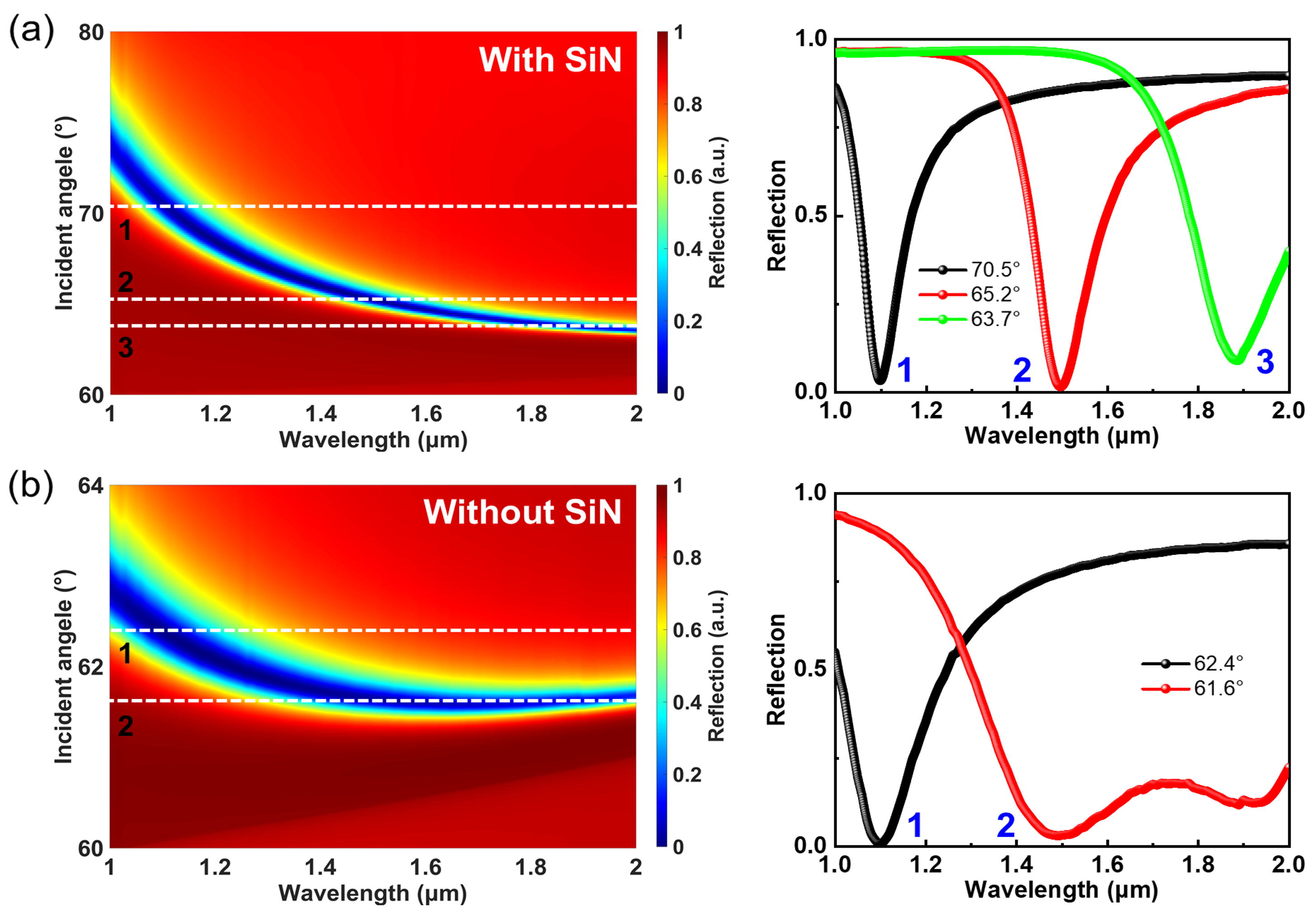
**

**Figure S5.** SP dispersion tuning. (a) Reflection spectra of a stack in Figure S4 in a Kretschmann configuration. (b) Reflection spectra of a stack removing the SiN layer from that in Figure S4.

1. **Optical and photoelectric characterization methods**

To measure the reflection spectra and the photoelectric response spectra, a collimated and programmable supercontinuum laser source (NKT Photonics A/S SC-400) together with an acousto-optic tunable filter (AOTF) was used for wavelength-dependent optical and photoelectric measurements. The linewidth of the AOTF is 6.4-19.8 nm within a wavelength range of 1100-2000 nm. A signal generator (VICTOR 2015H) was used to apply bias to LC. A source meter (Keithley 2636B, Keithley Instruments, Inc.) was used to collect the photoelectric signals. An optical power meter (Thorlabs PM100D) was used to measure the power of the incident light.

In experiment, at each bias on the LC layer, the wavelength of monochromatic light from the NKT laser source was tuned by an AOTF to scan through the interesting wavelength range. For the measurement of reflection spectra, both incident light (before entering the LC unit) and reflected light (after reflecting back from the LC unit) were recorded by a power meter, and the reflection at each wavelength (5 nm step in 1100-2000 nm) was obtained by normalizing the reflected light (*P*_out_) to the incident light (*P*_in_). For the measurement of photoelectric response spectra, the reflected light was recorded by the integrated PbS PD (photocurrent *I*) and the photoelectric response *R* can be calculated by *I*/*P*_in_.

1. **Photoelectric characterization of the PbS photodetector**

The PbS photodetector operates at a photoconductive mode and its I-V curve in a dark state is shown in Figure S6a. The response time is approximately 2 ms as shown in Figure S6b. The photoelectric response of the PbS photodetector covers a broad wavelength range with a responsivity over 1 A/W as shown in Figure S6c. A NKT supercontinuum laser together with an AOTF (900-2000 nm) and a tunable laser (2960-3040 nm) were used to collect the photoelectric response spectrum, while there is no applicable light source for the wavelength range of 2000-2960 nm.

**
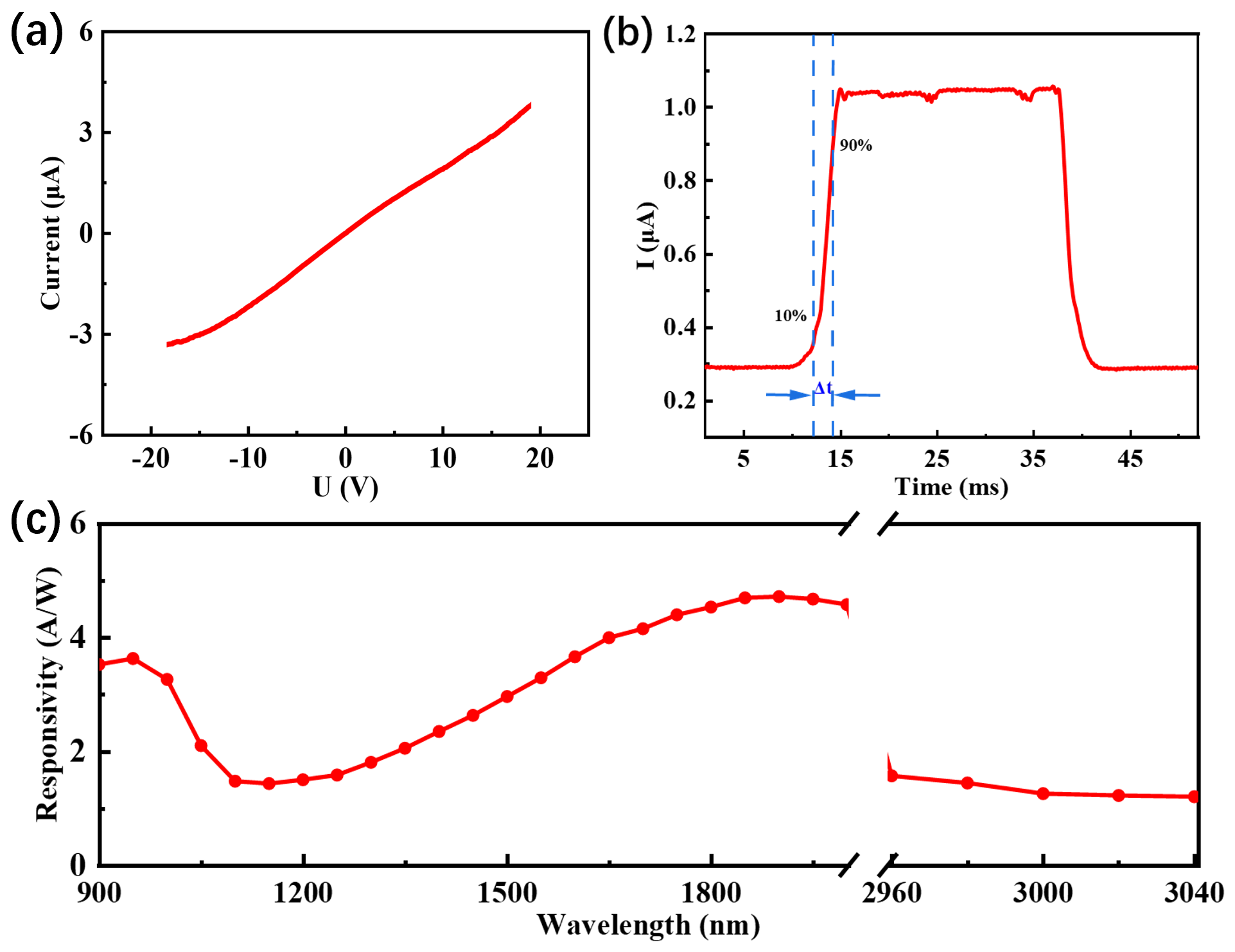
**

**Figure S6.** Photoelectric characterization of the PbS Photodetector. (a) Dark I-V curve. (b) Time-dependent photoelectric response curve. (c) Photoelectric response spectrum.

1. **Wavelength-dependent refractive index sensitivity**

In most cases, light modulation and optical sensing share similar mechanisms in terms of light-matter interaction. For light modulation, RI is usually tuned to change light transmission or optical resonance. For optical sensing, the variation of light transmission or optical resonance was usually used to detect the RI variation of the analytes. Prism-coupled SPR is well known for its high RI sensitivity. This RI sensitivity is not a fixed value but depends on the wavelength, which may have an influence on light modulation in a broad band. For a same prism-coupled SPR structure as mentioned in Supplementary S5, the RI sensitivity was calculated as shown in Figure S7. As seen, the sensitivity *S* = Δ*λ*/Δ*n* increases monotonically with the increasing wavelength, where Δ*n* is the change in RI of the material on the Au surface, and Δ*λ* is the shift of SPR. The sensitivity is approximately 2000 nm/RIU at λ=1000 nm, 10000 nm/RIU at λ=1480 nm, and over 38000 nm/RIU at λ=2000 nm, respectively. Therefore, with a relatively larger initial SPR wavelength during the reconfigurable photodetection experiment, the LC-based wavelength tuning range could be larger. It is expected that the wavelength tuning range could be larger if the tuning starts at a larger initial wavelength at a zero bias. For example, at an incident angle of 64.9°, the SPR wavelength is 1480 nm at 0 V and it shifts from 1480 nm to over 2000 nm with a bias less than 4.5 V as shown in Figure S8a. In contrast, the SPR shifts only 280 nm with a bias over 10 V in Figure 2, where the SPR wavelength is 1150 nm at 0 V. The calculated reflection spectra are shown in Figure S8b, which are consistent with the measured ones.

**Figure S7.** Wavelength-dependent RI sensitivity of the prism-coupled SPR.

**
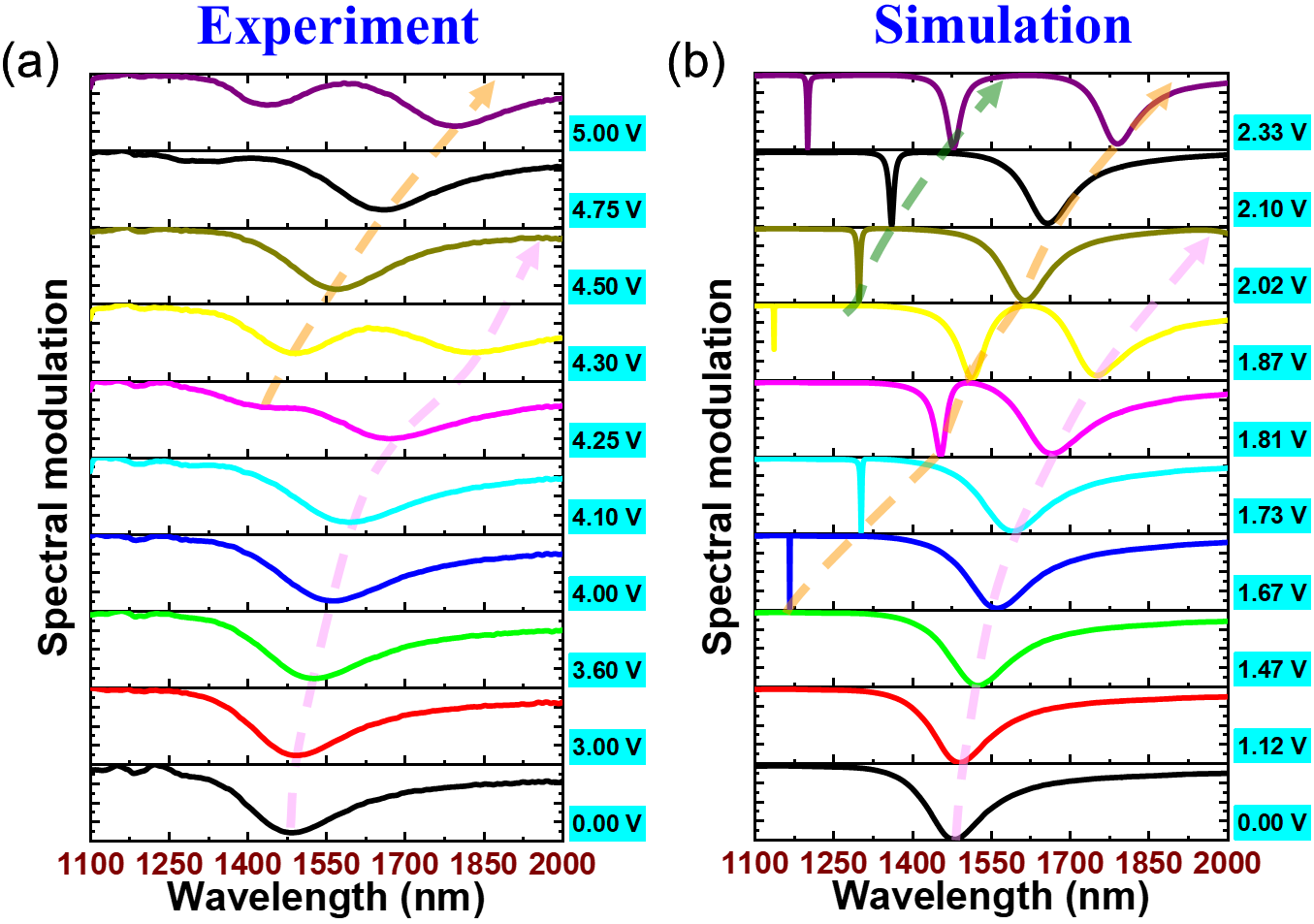
**

**Figure S8.** LC modulated reflection spectra. (a) Experiment and (b) Simulation results for an incident angle of 64.9° with a SPR wavelength of 1480 nm at 0 V.

1. **Spectral reconstruction**

For spectral reconstruction, a response matrix (***C****_M_*_×_*_N_*) of the reconfigurable PD is usually calibrated in advance, which consists of photoelectric responses *R*(*λ*,*U*) under *M* different monochromatic illumination (*λ*) at *N* different biases (*U*). In experiment, the photocurrent *I*(*λ*,*U*) of the reconfigurable PD was recorded firstly in sequence when the LC bias was continuously tuned for each monochromatic incident light. Then, each photoelectric responses *R*(*λ*,*U*) can be calculated as *I*(*λ*,*U*)/*P*_in_(*λ*), where *P*_in_(*λ*) is the power of monochromatic illumination recorded by a power meter. All photoelectric responses *R*(*λ*,*U*) form a calibration matrix (***C****_M_*_×_*_N_*). For example, in Figure 4a a response matrix (***C***_101×21_) was recorded at a wavelength step size of 5 nm (NKT light source) in a wavelength range of 1250-1750 nm and a bias step of 50 mV in a voltage range of 4.8-5.8 V. Similarly, for Figure 4b a response matrix (***C***_13×9_) was recorded at a wavelength step size of 5 nm (NKT light source) in a wavelength range of 1520-1580 nm and a bias step of 10 mV in a voltage range of 5.41-5.49V, and for Figure 4c a response matrix (***C***_61×9_) was recorded at a wavelength step size is 1 nm (Santec light source) in a wavelength range of 1520-1580 nm and a bias step is 10 mV in a voltage range of 4.96-5.04 V. A time step of 100 ms was used. For incident light with a spectrum *S*(*λ*), the output photocurrents *I_i_* (*i* = 1, 2, …, *N*) were recorded as the LC bias was continuously tuned,

$I_{i}=\int_{\lambda_{1}}^{\lambda_{2}} S(\lambda)\times C_{M\times i}(\lambda)d\lambda$ (equ. S2)

The Equ. S2 can be further rewritten in a matrix form as

$\boldsymbol{I}=\boldsymbol{C}\cdot\boldsymbol{S}$ (equ. S3)

Then, a software CVX for specifying and solving convex program^1,2^ was used for solving this equation to reconstruct the original spectrum *S*(*λ*).

$\min\left\| \boldsymbol{I-C}\cdot\boldsymbol{S} \right\|_{2}^{2}$ subject to ***S*** > 0. (equ. S4)

Figure S9 shows the spectral reconstruction result of the transmission spectrum of a thick piece of PMMA in a broad band (1100-1500 nm). During measurement, the AOTF was disconnected from the NKT light source and removed from the light path.


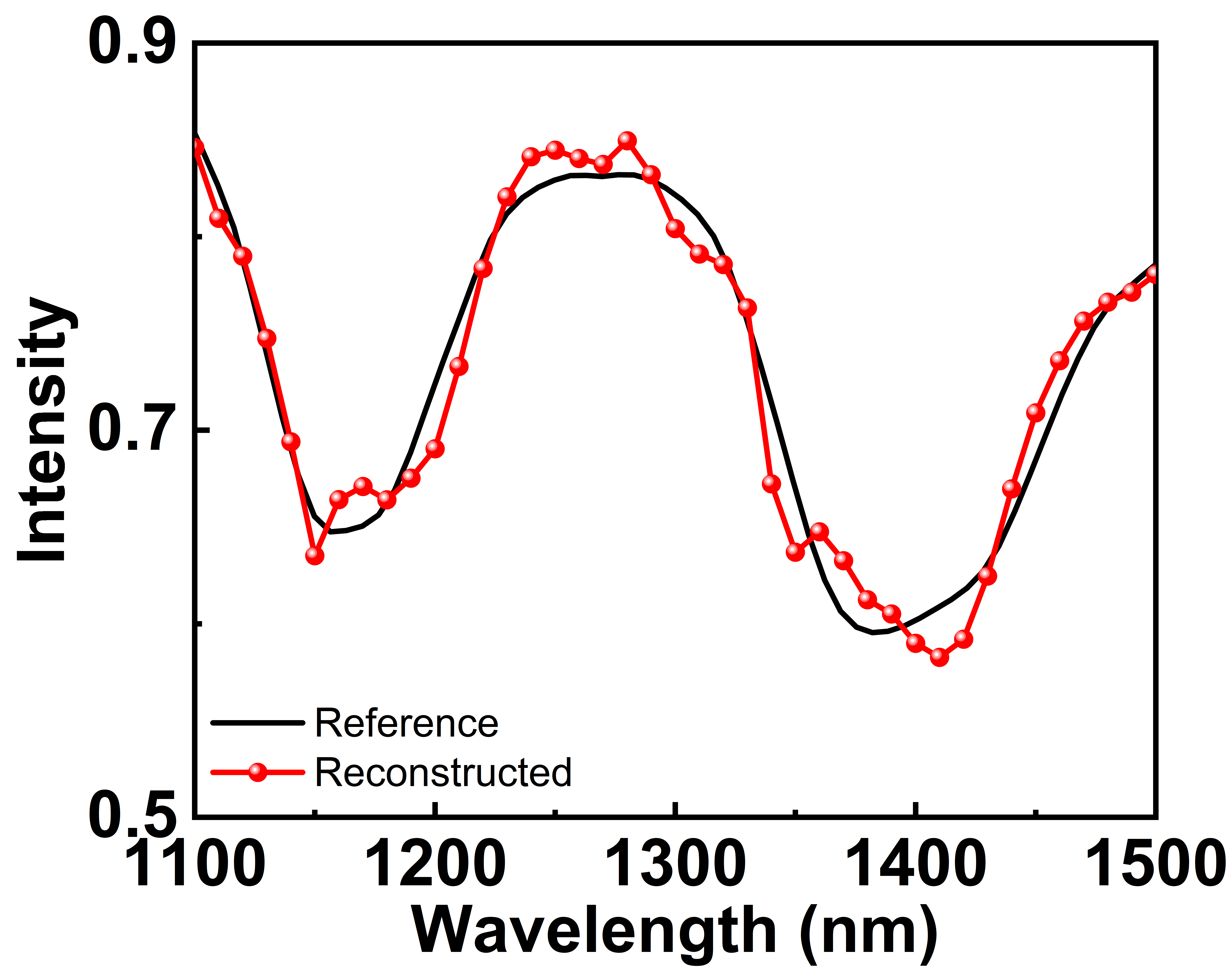


**Figure S9**. Reconstructed transmission spectrum of a thick PMMA sample in a broad band. The measured result with a commercial spectrometer is shown for comparison.

1. **Plastic sorting**

The photoelectric response spectra of the reconfigurable PD at 44 voltages (6.1-17.5V) were plotted in Figure S10a-S10d under the broadband illumination of the NKT supercontinuum laser source (without an AOTF). For the 1^st^ (6.1-8.1V), 2^nd^ (8.2-9.2V), 3^rd^ (9.4-10.4V) and 4^th^ (13.5-17.5V) 11-voltage set, the SPR appears in Band 1 (1118-1218 nm), Band 2 (1224-1324 nm), Band 3 (1330-1430 nm) and Band 4 (1620-1720 nm) respectively, which dominates the main photocurrent modulation. The transmitted light through different plastic samples under the same illumination was recorded by the reconfigurable PD at the same 44 voltages as shown in Figure S10e-S10h. These output photocurrents contain spectral absorption related features of various plastics.

Linear discriminant analysis (LDA) method^3^ was used for classification of the plastics based on the measured photocurrents. LDA is a statistical method widely used for classification and dimensionality reduction by finding the optimal linear combination of features to well separate different classes. In this work, both recorded photocurrents and ground truths of the plastic categories were used as input features and labels to build the LDA classification model. 420 photoelectric responses collected from different samples were used to train the model via a 5-fold cross-validation method^4^. In total 105 plastic samples different from the training samples were used for testing.

**
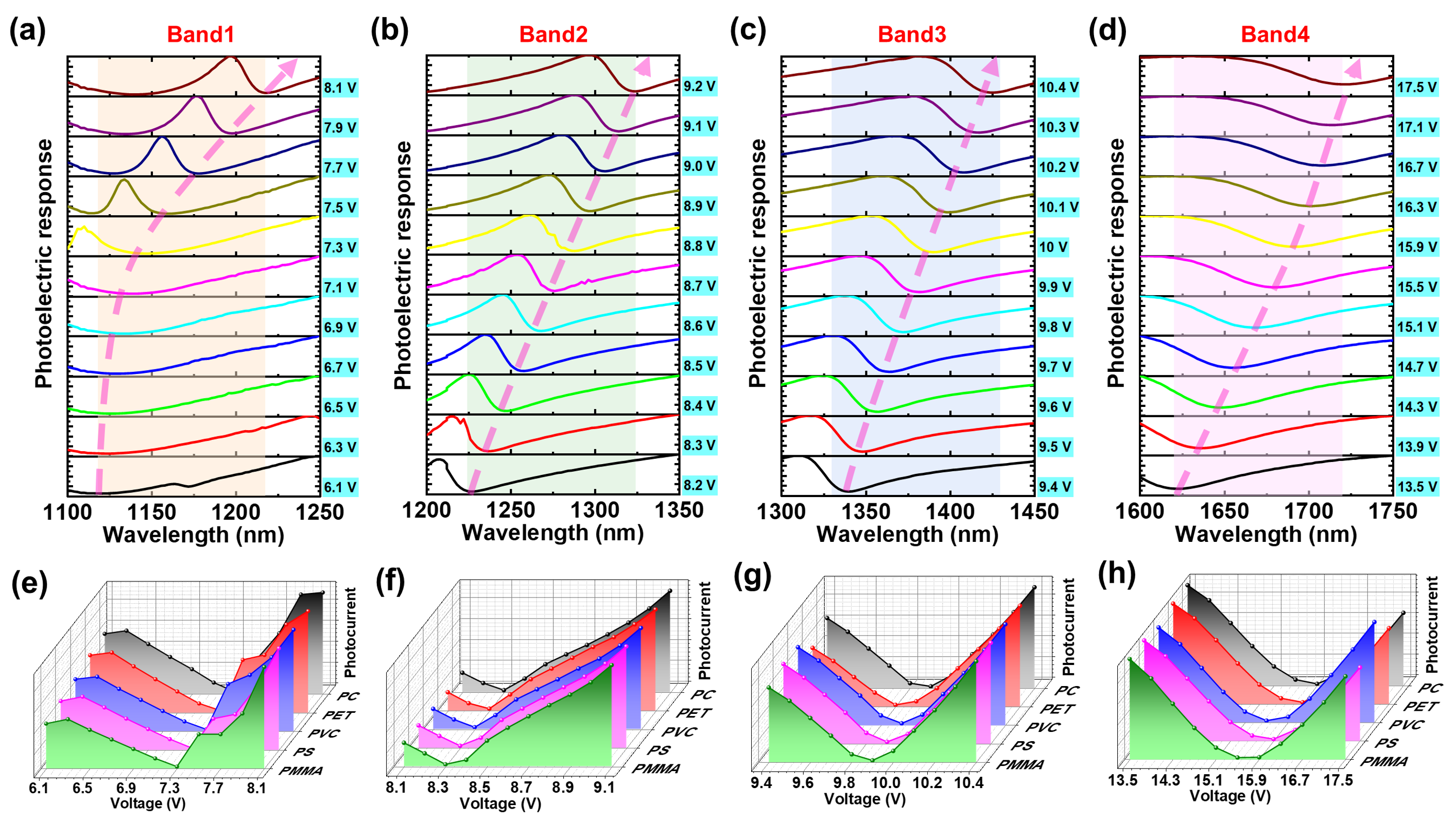
**

**Figure S10.** Photoelectric responses in the plastic sorting experiment. (a)-(d) The photoelectric response spectra of the reconfigurable PD at 44 voltages (6.1-17.5V) were plotted under the broadband illumination of the NKT supercontinuum laser source (without an AOTF). (e)-(h) The photoelectric responses for the same illumination through five plastic samples at the same 44 biases.

**Table S2. 44 biases in the plastic sorting experiment**

| **Band1** | **Band2** | **Band3** | **Band4** | **Figure 5g** |
| --- | --- | --- | --- | --- |
| 6.1 V | 8.2 V | 9.4 V | 13.5 V | 7.7 V |
| 6.3 V | 8.3 V | 9.5 V | 13.9 V | 7.9 V |
| 6.5 V | 8.4 V | 9.6 V | 14.3 V | 8.1 V |
| 6.7 V | 8.5 V | 9.7 V | 14.7 V | 8.2 V |
| 6.9 V | 8.6 V | 9.8 V | 15.1 V | 13.5 V |
| 7.1 V | 8.7 V | 9.9 V | 15.5 V | 14.3 V |
| 7.3 V | 8.8 V | 10.0 V | 15.9 V | 14.7 V |
| 7.5 V | 8.9 V | 10.1 V | 16.3 V | 15.1 V |
| 7.7 V | 9.0 V | 10.2 V | 16.7 V | 16.7 V |
| 7.9 V | 9.1 V | 10.3 V | 17.1 V | 17.1 V |
| 8.1 V | 9.2 V | 10.4 V | 17.5 V | 17.5 V |

**References**

[1] CVX Research, Inc. CVX: Matlab software for disciplined convex programming, version 2.0. https://cvxr.com/cvx, April 2011.

[2] M. Grant and S. Boyd. Graph implementations for nonsmooth convex programs, Recent Advances in Learning and Control (a tribute to M. Vidyasagar), V. Blondel, S. Boyd, and H. Kimura, editors, pages 95-110, Lecture Notes in Control and Information Sciences, Springer, 2008.

[3] Tharwat A, Gaber T, Ibrahim A, Hassanien AE. Linear discriminant analysis: A detailed tutorial. *Ai Commun* **30**, 169-190 (2017).

[4] Berrar D. Cross-validation. *Encyclopedia of bioinformatics and computational biology* **1**, 542-545 (2019).
